# Liposomal Formulations to Modulate the Tumour Microenvironment and Antitumour Immune Response

**DOI:** 10.3390/ijms19102922

**Published:** 2018-09-26

**Authors:** Roger Gilabert-Oriol, Gemma M. Ryan, Ada W.Y. Leung, Natalie S. Firmino, Kevin L. Bennewith, Marcel B. Bally

**Affiliations:** 1 Department of Experimental Therapeutics, British Columbia Cancer Research Centre, Vancouver, BC V5Z 1L3, Canada; gemmaryan3@gmail.com (G.M.R.); aleung@bccrc.ca (A.W.Y.L.); 2 Cuprous Pharmaceuticals Inc., Vancouver, BC V6N 3P8, Canada; 3 Department of Chemistry, University of British Columbia, Vancouver, BC V6T 1Z1, Canada; 4 Department of Integrative Oncology, British Columbia Cancer Research Centre, Vancouver, BC V5Z 1L3, Canada; nfirmino@bccrc.ca (N.S.F.); kbennewi@bccrc.ca (K.L.B.); 5 Department of Pathology and Laboratory Medicine, University of British Columbia, Vancouver, BC V6T 2B5, Canada; 6 Faculty of Pharmaceutical Sciences, University of British Columbia, Vancouver, BC V6T 1Z3, Canada; 7 Centre for Drug Research and Development, Vancouver, BC V6T 1Z3, Canada

**Keywords:** liposomes, tumour microenvironment, tumour vasculature, tumour stroma, tumour-infiltrating lymphocytes, immunogenic cell death, radiotherapy, doxorubicin, irinotecan, paclitaxel, mifamurtide

## Abstract

Tumours are complex systems of genetically diverse malignant cells that proliferate in the presence of a heterogeneous microenvironment consisting of host derived microvasculature, stromal, and immune cells. The components of the tumour microenvironment (TME) communicate with each other and with cancer cells, to regulate cellular processes that can inhibit, as well as enhance, tumour growth. Therapeutic strategies have been developed to modulate the TME and cancer-associated immune response. However, modulating compounds are often insoluble (aqueous solubility of less than 1 mg/mL) and have suboptimal pharmacokinetics that prevent therapeutically relevant drug concentrations from reaching the appropriate sites within the tumour. Nanomedicines and, in particular, liposomal formulations of relevant drug candidates, define clinically meaningful drug delivery systems that have the potential to ensure that the right drug candidate is delivered to the right area within tumours at the right time. Following encapsulation in liposomes, drug candidates often display extended plasma half-lives, higher plasma concentrations and may accumulate directly in the tumour tissue. Liposomes can normalise the tumour blood vessel structure and enhance the immunogenicity of tumour cell death; relatively unrecognised impacts associated with using liposomal formulations. This review describes liposomal formulations that affect components of the TME. A focus is placed on formulations which are approved for use in the clinic. The concept of tumour immunogenicity, and how liposomes may enhance radiation and chemotherapy-induced immunogenic cell death (ICD), is discussed. Liposomes are currently an indispensable tool in the treatment of cancer, and their contribution to cancer therapy may gain even further importance by incorporating modulators of the TME and the cancer-associated immune response.

## 1. Introduction

Cancer is a leading cause of death worldwide. In 2018, cancer statistics in the United States predicted more than 1.7 million new cancer cases and over 600,000 cancer-related deaths [1]. Various treatment strategies are available to help the patients and manage the disease, depending on the type and stage of the disease at diagnosis. This includes surgery to remove the tumour bulk, cytotoxic chemotherapy and radiotherapy to selectively kill the rapidly dividing and partially impaired cancer cells, targeted therapies directed towards specific genetic drivers of cancer, and immunotherapy to stimulate the innate and acquired immune system against malignant cells [2]. The number of cancer survivors has increased in recent decades, partly due to advances in early detection, but also because of the improved treatment outcomes from new therapeutic strategies [3]. However, despite this large repertoire of treatments, cancer cells develop resistances to therapies [4], and disseminate from the primary tumour to distant sites forming metastases [5,6] which ultimately kill the patient. New treatments, consisting of novel combinations of existing therapies and new innovative therapeutics, are urgently needed, particularly in the case of metastatic disease.

Tumours have been historically perceived as groups of cells with deregulated growth that proliferate without control and, at later stages, metastasise. However, tumours are not exclusively cells behaving independently and are, instead, complex structures of malignant cells that constantly interact with the surrounding microenvironment [7] and change because of accumulating mutations [8]. The microenvironment is a key factor during cancer development and often has tumour-promoting functions [9]. The main components of the tumour microenvironment (TME) are non-malignant cells that secrete cytokines, chemokines, growth factors, inflammatory and matrix remodelling enzymes to build the modified tumour stroma, as well as blood and lymphatic vasculature [10]. These non-malignant cells have also a profound effect on the efficacy of anticancer therapies, and include cancer-associated fibroblasts, vascular endothelial cells, and cells of the immune system, such as tumour-infiltrating lymphocytes, tumour-associated macrophages, and myeloid-derived suppressor cells [11]. Common non-cellular features of the TME are hypoxia, nutrient deprivation, low pH, and high interstitial fluid pressure [12].

Drug candidates have been developed to target the components of the TME in order to overcome acquired resistances, prevent metastasis of cancer cells, and improve therapeutic efficacy [13]. However, many of these compounds are of hydrophobic nature, resulting in poor aqueous solubility and may be rapidly eliminated, poorly adsorbed if given orally, and/or may present undesired biodistribution. Liposomes are a well-described drug delivery system that has transitioned to clinical applications with proven capabilities that can overcome these problems [14]. Liposomes are spherical lipid vesicles, typically with a mean diameter of 100 nm and composed of a phospholipid bilayer with or without cholesterol. They have an aqueous core, and the bilayer itself creates a hydrophobic region [15]. In addition to the encapsulation of hydrophobic drugs, extension of blood circulation time, and increase in drug exposure to the tumour tissue, liposomes also facilitate the distribution of the associated drug to the TME [16]. Although heterogeneous, passive accumulation of liposomal formulations occurs through the enhanced permeability and retention (EPR) effect, a phenomenon that is based on the prolonged circulation of liposomes, the leaky vasculature surrounding the tumour that allows selective extravasation of liposomes, and the impaired tumour-associated lymphatic system, that prevents the elimination of vesicles from the tumour tissue [17].

There is a great potential for liposomal formulations to enhance the delivery of compounds with potential anticancer activity—compounds synthesised to modulate the TME and reactivate the tumour-associated immune response. In this review, the main components of the TME and tumour-associated immune response are described, as well as therapeutic approaches to modulate them to achieve improved outcomes in patients with cancer. A search was conducted to identify liposomal formulations that can alter the TME and the immune system. An emphasis was placed on regulatory approved liposomal formulations. The focus, for this reason, is only on liposomal formulations that passively accumulate to regions of the tumour where they can affect the TME. Liposomes actively targeted through tumour-specific ligands or antibodies are described in other reviews [18,19], but at the moment, are not suitable for development and clinical testing as lead candidates, despite a great deal of effort. Furthermore, the use of liposomal formulations to provide innovative approaches to deliver agents capable of inducing immunogenic cell death (ICD) is considered. More specifically, the combination of liposomal drugs with radiotherapy is also discussed with regards to potential enhancement of the immunogenicity of dying cancer cells. The exciting possibilities of using liposomes for the modulation of the TME and tumour-associated immune responses, as well as how cancer patients may benefit from these novel formulations, is discussed. Optimal treatment outcomes will only be achieved through use of combinations and liposomes are, at the moment, the best strategy to prepare drug combination products, as exemplified by the recent approval of Vyxeos™, the first combination product designed to specifically take advantage of the ability of liposomes to coordinate delivery of multiple drugs.

## 2. The Tumour Microenvironment

Tumour cells originate from the accumulation of genetic and epigenetic alterations [20]. These changes affect gene expression, result in the deregulation of normal cell function, and contribute to development of treatment resistance. Malignant cells are, therefore, capable of sustaining chronic proliferation and resisting cell death by activating and suppressing a range of defined cellular mechanisms [21]; mechanisms that allow tumour cells to metastasise and prevent effective removal of the cells by the immune system. As indicated already, tumour cells do not proliferate in isolation from normal tissues but evolve by interacting with progressively altered benign tissue. Non-malignant cells that secrete proteins and provide nutrients are essential during the initiation and progression of carcinogenesis. This community of cancerous and non-malignant, modified stromal cells forms the tumour microenvironment (TME) [22]. In addition to supporting the progression of tumour cells, the microenvironment also influences their development, protects them from the immune system, and mediates the transformation to more aggressive malignancies, resistance to therapies and metastasis [23]. The key conditions of the TME are listed in Table 1. The main properties and components of the TME and how they influence the development of solid tumours are described in detail in the following sections. Therapeutic strategies that target the different parts of the TME resulting in inhibition of tumour growth are then discussed.

### 2.1. Hypoxia and Acidity in the Tumour Stroma

A primary characteristic of the TME is the presence of hypoxic regions that are less oxygenated and less nutritious relative to normal physiological conditions [24]; an effect caused by poor/limited blood flow. During the first stages of tumour development, solid tumours occupy small volumes, allowing cancer cells to receive the necessary nutrients and physiological levels of oxygen through the normal vasculature. As tumours proliferate, they progressively increase in volume so that while some tumour cells remain close to the blood vessels and have access to enough oxygen to continue proliferating (within 100 µm to 200 µm of blood vessels), other tumour cells find themselves distant from the blood vessels [25]. Oxygen is not able to diffuse at concentrations high enough to allow active proliferation. Under these hypoxic conditions, tumour cells undergo hypoxia-induced changes in gene expression that provide survival advantages, such as those linked to the expression of the hypoxia inducible factor (HIF) system [26]. These changes in gene expression result in the suppression of apoptosis (downregulation of BID and BAX), support of autophagy (upregulation of MAP1LC3B and ATG5), and allow the cells to switch to anabolic metabolism [27]. Tumour cells under hypoxic conditions acquire energy mainly through fermentative metabolism based on anaerobic glycolysis and consequently co-secrete higher amounts of protons and lactate to the extracellular space [28]. Protons are liberated to the tumour interstitium by proton pumps and metabolic transporters, such as the H^+^-ATPases, the Na^+^–H^+^ exchanger NHE1, and the monocarboxylate-H^+^ efflux cotransporters MCT1 and MCT4 [29]. A direct consequence of this altered metabolism in cells within hypoxic regions of solid tumours is the drop in pH. While physiological pH in healthy tissue is 7.2–7.4, the acidic environment of tumour cells can reach lower values in the range of 6.5–6.9 [30]. Acidity in the tumour tissue has a range of effects, including induction of signal transduction pathways, selective gene expression, activation of extracellular metalloproteinases, as well as immunosuppression [31]. This aids in the development of treatment resistance, promotes metastasis, and ensures the cells remain fit for survival [30].

From a therapeutic point of view, hypoxia, acidity, and their corresponding modifications in the tumour stroma, can be exploited by nanomedicines, such as liposomes, with an associated drug candidate, to better target the region of cancer growth and widen the therapeutic window. Liposomes that passively accumulate at the tumour site have been designed to activate prodrugs in response to low levels of oxygen and liberate high concentrations of the therapeutic agent in the hypoxic microenvironment. For example, liposomes containing the hypoxia-activated prodrug AQ4N improved cancer treatment outcomes in vivo, preclinically, after photodynamic therapy [32]. Liposomes have also been designed to lose integrity and release their cargo at low pH in the acidic regions of the tumour. As an example, pH-sensitive liposomes containing doxorubicin demonstrated antitumour activity in mice bearing gliomas [33].

### 2.2. Tumour Neovasculature and Inflammation

Tumours may form new, irregular, and leaky blood vessels that allow the trafficking of tumour-infiltrating lymphocytes (TILs) into the tumour tissue, as if the tissue was undergoing chronic inflammation [34]. Hypoxic conditions and nutrient deprivation (previous section) induce the secretion of cytokines and growth factors such as vascular endothelial growth factor A (VEGFA), stromal-derived factor 1 alpha (SD1α), tumour necrosis factor alpha (TNF-α), interleukin (IL)-1β, and IL-6) from tumour cells to trigger the formation of new blood vessels (neovascularisation or angiogenesis) and inflammation [35]. In comparison to normal endothelial cells, tumour-associated endothelial cells show fibroblast-like properties after undergoing an endothelial-to-mesenchymal transition, downregulating the endothelial cell marker CD31 and upregulating the tumour-associated fibroblast markers fibroblast-specific protein 1 (FSP1) and alpha-smooth muscle actin (alpha-SMA) [36].

Angiogenesis occurs by different physiological processes, such as sprouting angiogenesis, intussusceptive microvascular growth, vascular co-option, and vasculogenic mimicry [37]. All these angiogenic mechanisms occur during the development of solid tumours and, as a result, normal vasculature is transformed into abnormal blood vessels. The newly created tumour blood vessels are distinct from normal vasculature: they are irregular with a discontinuous endothelium and a leaky basal membrane. This allows components of the blood to readily extravasate into the tumour interstitium and facilitates the transmigration of lymphocytes, which is mediated by intracellular adhesion molecule 1 (ICAM-1), vascular cell adhesion molecule 1(VCAM-1), and E-selectin [38]. Several chemokines produced by both cancer and stroma cells (CCL2, CCL3, CCL4, CCL5, CXCL9, and CXCL10) also serve as chemoattractants to lymphocytes [39]. These, along with a permeable neovasculature and inflamed tumour tissue, allow the infiltration of lymphocytes and the beginning of an antitumour immune response.

The leaky blood vessels can be leveraged by certain types of treatments. For instance, high molecular weight drugs and nanoparticles can passively accumulate at the inflamed tumour tissue via the EPR effect [17]. Alternatively, other therapeutic strategies are used to downregulate tumour neovascularisation, in order to reduce the nutrient supply and subsequent growth of tumour cells. This can be achieved by using conventional chemotherapeutics given metronomically [40], or even liposomes which have been reported to have suppressive effects on the tumour vasculature [41]. There are also targeted agents to the vascular endothelial growth factor (VEGF) family members and their cognate receptors. The targeted agents, which are approved for treating a wide range of cancer modalities, are small molecular weight drug inhibitors (sunitinib, sorafenib, pazopanib, vandetanib, axitinib, aflibercept, regorafenib, nintedanib, and lenvatinib) and therapeutic antibodies (bevacizumab and ramucirumab) [42].

### 2.3. Barriers in the Tumour Stroma Preventing Infiltration of Lymphocytes

Tumour cells may express neoantigens that can be presented to the immune system, triggering a host immune response [43]. Although tumour cells may be immunogenic per se, cancer patients often lack TILs and the associated immune-mediated tumour rejection [44]. In fact, lymphocyte infiltration can be prevented by physical barriers, such as a dense extracellular matrix in the tumour stroma, non-permissive vasculature, and lack of inflammation.

Tumour-associated fibroblasts contribute to remodel the tumour’s extracellular matrix. They are permanently activated and can be differentiated from normal fibroblasts by the production of alpha-SMA and increased expression of fibroblast activation protein (FAP) [45]. Tumour-associated fibroblasts secrete increased quantities of structural proteins (collagen and elastin), specialised proteins (fibronectin, fibrillin, and laminin), and proteoglycans, in comparison to fibroblasts in normal tissue [46]. The deposition of these molecules, especially collagen and fibronectin, increase the rigidity of the extracellular matrix and act as a physical barrier that decreases tumour perfusion and prevents lymphocyte infiltration. Furthermore, excessive connective tissue, in conjunction with vessel abnormalities and contraction of the interstitial space, results in high interstitial fluid pressure that limits transcapillary transport of therapeutic agents [47]. Tumour-associated fibroblasts also secrete cytokines and growth factors that promote proliferation of cancer cells and resistance to therapeutic agents and metastasis [46].

There is increasing evidence that cells derived exclusively from the adipose tissue (cancer-associated adipocytes) are also involved in remodelling the extracellular matrix [36]. Cancer-associated adipocytes have been associated with enhanced production of matrix metalloproteinase-11 (MMP-11) and pro-inflammatory cytokines (IL-1β and IL-6) [48], as well as increased degradation of lipids, to provide additional energy to malignant cells at the tumour front [49]. Further research is required to elucidate the exact contribution of cancer-associated adipocytes in remodelling the extracellular matrix and the TME. Of note, other cell types, such as the same cancer cells, immune cells, and epithelial cells in late-stage tumours, contribute to remodelling the extracellular matrix, as well [50].

As a result of the crosstalk between stromal cells and cancer cells, tumours may exhibit poor perfusion and lack TILs. However, therapeutic strategies have been proposed to induce inflammation in the tumour tissue and trigger lymphocyte infiltration. Intratumour administration of interferon-β, introduction of the TNF superfamily member LIGHT, inhibition of specific oncogenic pathways, and local radiotherapy exhibited improved lymphocyte infiltration in preclinical tumour models [51]. Anticancer vaccines, such as the clinically approved sipuleucel-T, stimulated the immune system against tumours [52]. Furthermore, chemotherapy potentiated the intratumour recruitment of antigen-presenting cells and mediated anticancer immune response by T lymphocytes [53]. Both radiation and certain types of chemotherapy (e.g., anthracyclines, mitoxantrone, oxaliplatin, and bortezomib) are able to trigger immunogenic cell death (ICD) and engage the host immune system against a tumour that would otherwise evade immune detection [54]. Combination regimens of chemotherapy and radiotherapy [55], as well as liposomal formulations of chemotherapeutic drugs [56], have been proposed to enhance ICD. The concept of ICD and therapeutic strategies to achieve ICD effects are further discussed in Section 3, Section 4.5 and Section 4.6 below.

### 2.4. Immune Cells in the TME

As previously mentioned, genetic mutations that occur in cancer cells result in the generation of neoantigens that can be recognised by the host immune system. In the context of leaky neovasculature and inflammation, immune cells are able to penetrate into the TME and initiate an anticancer immune response. In accordance with this, the presence of TILs, particularly cytotoxic CD8^+^ T lymphocytes, are correlated with better therapeutic outcomes, and it has been proposed that the presence of these cells can serve as a positive prognostic marker in colorectal cancer [57]. Analogous observations were made in breast cancer [58], melanoma [59], and gastrointestinal stromal tumours [60].

The priming of the immune response is a mechanism in which initially DNGR-1^+^ dendritic cells (first characterised in mice as CD8α^+^ dendritic cells) acquire antigens from dying tumour cells and target the antigens for cross-presentation via the class I major histocompatibility complex (MHC) processing pathway [61,62]. Dendritic cells release a series of chemokines and cytokines that activate CD8^+^ T-cells and stimulate their infiltration in the tumour. Secretion of type I interferon induced by the STING pathway after detection of extracellular DNA derived from dying tumour cells also seems to have a critical functional role during the antigen cross-presentation to CD8^+^ T-cells [51]. Cytotoxic CD8^+^ T lymphocytes are then able to recognise cancer cells and eliminate them by releasing perforin and granzymes. Natural killer cells detect and eliminate cancer cells that are deficient in class I MHC, present upregulated stress ligands or are labelled with specific antibodies against tumour neoantigens [63].

**Figure 1:**
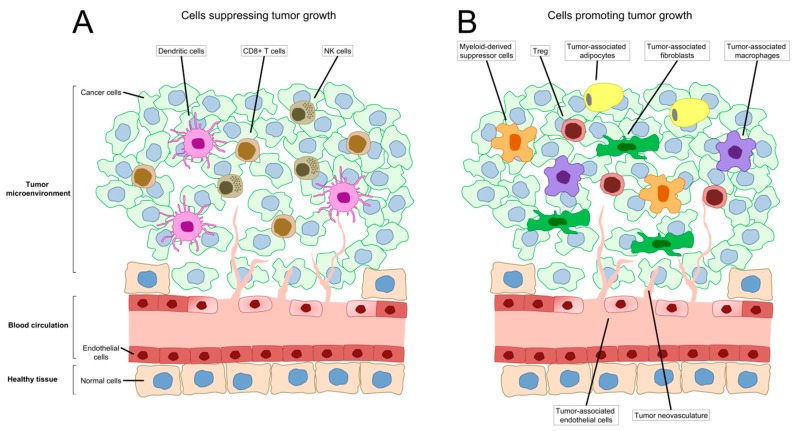
Cells in the tumour microenvironment that modulate tumour growth. (**A**) Cells suppressing tumour growth include dendritic cells responsible for acquisition of antigens from dying tumour cells and cross-presentation; cytotoxic CD8^+^ T lymphocytes that recognise cancer cells with the tumour-associated antigens and eliminate them; and NK cells that alternatively detect cancer cells deficient in MHC I, presenting stress signatures or are opsonised and, likewise, deplete them. (**B**) Cells promoting tumour growth include tumour-associated endothelial cells and the corresponding tumour neovasculature; tumour-associated fibroblasts and tumour-associated adipocytes that mainly remodel the tumour’s extracellular matrix; and T_regs_, tumour-associated macrophages and myeloid-derived suppressor cells all involved in various mechanisms of immune suppression.

The presence of all the elements necessary to trigger a host anticancer immune response (tumour-associated antigens, cross-presentation of antigens by DNGR-1^+^ dendritic cells, and infiltration of CD8^+^ T-cells and NK cells), as suggested above, does not always result in an effective immune response against the tumour. This is mainly due to the aberrant upregulation of a series of immunosuppressive pathways that are necessary for the normal function of the immune system. Several inhibitory pathways have been extensively described, including the programmed death ligand PD-L1/PD-1 pathway [64], expression of the cellular enzyme indoleamine-2,3 dioxygenase (IDO) [65], presence of regulatory T-cells (T_reg_) [66,67,68], and T-cell anergy [69]. Furthermore, there are other groups of immune cells present in the TME that have been studied for their tumour promoting and immune suppressor roles (Figure 1), for example tumour-associated macrophages and myeloid-derived suppressor cells [70]. Tumour-associated macrophages seem to lose the capacity of eliminating malfunctioning cells through a process of polarisation (from M1 classically activated to M2 alternatively activated subtype) and contribute immunosuppression by secreting IL-10 and TGF-β [71,72]. Myeloid-derived suppressor cells are immature myeloid cells closely related to neutrophils and monocytes that induce suppression of the immune system through the depletion of arginine by the cellular enzyme arginase (ARG1), nitrosylation (iNOS), and other mechanisms [73].

The blockade of these inhibitory pathways or cell subtypes was conceived as a strategy to block immunosuppression and to reactivate the immune system against the tumour. This has been successfully achieved in the clinic by therapeutic antibodies that are targeted to the ligand PD-L1 (atezolizumab, avelumab, durvalumab), the receptor PD1 (nivolumab, pembrolizumab), and the receptor CTLA-4 (ipilimumab) [74]. The administration of interleukin IL 2 demonstrated activation of the immune system and, especially, T-cells and NK cells in metastatic renal cell carcinoma and melanoma [75]. An alternative strategy, that recently received clinical approval, is to increase the number of tumour-infiltrating lymphocytes by administrating chimeric antigen receptor (CAR) T-cells directed against antigens expressed on cancer cells [76]. This modality of therapy consists of the isolation of T-cells from patients, expansion of T-cells which are genetically engineered to express CARs ex vivo and, then, injection of the modified T-cells back to patients. The two approved therapies so far are both targeted against CD19 (tisagenlecleucel and axicabtagene ciloleucel) [77]. Treatments described above may be used rationally, in a combination setting, to achieve stronger stimulation of the immune system against cancer cells and synergistic effects. This will be needed because in solid tumours, the CAR T-cell strategy appears to be more challenging. The use of rationally designed combinations will likely require the use of nanotechnology to ensure that the associated compounds are delivered to the right place so that they can impact the right cell populations, including tumour cells and host derived cells required to trigger immune responses.

## 3. Immunogenic Cell Death

In recent years, immunotherapy has garnered significant attention as it has been achieving great success in the clinic. This is illustrated by treatment approaches, such as blocking immune checkpoints with inhibitors that interfere with the PD-1/PD-L1 interaction to overcome immune suppression, or engineering patient T-cells to recognise and attack cancer cells through CAR-T therapy [78,79]. Generally, immunotherapy involves harnessing the host immune system to generate an antitumour response with the goal of completely eradicating cancer cells and generating a long-term antitumour immunity to achieve a cure [78,79]. Although tumour regression and long-term cancer-free statuses have been achieved in a considerable number of clinical trials involving immunotherapy, not all patients respond to these treatments [80]. The clinical benefits and limitations of the cytotoxic effects of conventional chemotherapy are well-known. However, it is only in recent years that the research community is recognising the immune-modulating capacity of some of these drugs and their potential to contribute with enhanced treatment outcomes when used as immunotherapeutics. These agents can, under the right circumstances, convert tumour cells into “therapeutic vaccines” through induction of immunogenic cell death (ICD), or can directly stimulate the immune system through promotion of immune cell maturation and activation, or inhibition of immunosuppressive cells, such as regulatory T-cells [80,81,82,83]. There is increasing interest in utilising chemotherapeutics to induce ICD which can generate long-term immunological memory, thus providing antitumour immunity against future cancer re-challenge [80,82,84]. Induction of ICD has, therefore, emerged as an exciting area of research in the development of novel immunogenic treatments to manage aggressive, recurrent, and metastatic cancers [80,84]. Importantly, existing information about immune cells within the TME, and compounds/drugs that have the potential to induce ICD, allows one to speculate about the design and use of nanomedicines specifically designed to induce ICD.

### 3.1. The Current Understanding of ICD and Its Underlying Molecular Mechanisms

ICD is a form of cell death where dying tumour cells emit signals known as damage-associated molecular patterns (DAMPs), leading to release of pro-inflammatory cytokines, activation of innate immune cells such as dendritic cells (DCs) and macrophages and, ultimately, stimulation of the adaptive immune response [85,86]. Currently, ICD is characterised by a spatiotemporally-defined combination of molecular signatures: (1) the pre-apoptotic expression of endoplasmic reticulum (ER) calreticulin (CRT) and heat shock proteins (HSPs) HSP70 and HSP90 on the cell surface, (2) the secretion of ATP, and (3) the secretion of nuclear high mobility group box 1 (HMGB1) [86,87]. Activation of an ER stress response is also required to successfully induce ICD. This ER stress response appears to be critical for the exocytosis of CRT to the cell surface, where it serves as an “eat me” signal for macrophages and DCs [88,89,90,91]. Many ICD inducers are known to generate reactive oxygen species (ROS). While ROS generation appears to contribute to the oxidative stress that could lead to CRT exposure, some studies suggest that ROS generation alone is not sufficient to induce ICD. Calcium leakage from the ER may be crucial to generating additional ROS from the mitochondria in order to reach the threshold for translocation of CRT to the outer leaflet of the plasma membrane [91]. On the other hand, the presence of HSPs on the cell surface allows the formation of tumour antigen–HSP complexes which are more readily processed by DCs for antigen presentation to T-cells [87].

As indicated above, a hallmark of ICD is the pre-mortem secretion of ATP. ICD-associated release of ATP involves a complex crosstalk among signalling molecules from multiple distinct cellular processes: autophagy, lysosomal exocytosis, apoptosis, membrane blebbing, and plasma membrane permeabilisation [83]. Upon treatment with ICD inducers, lysosomal ATP is redistributed to autolysosomes through an autophagy-dependent process, and is then translocated to the plasma membrane through lysosomal exocytosis. Apoptosis-related caspase activation is then required to mediate cellular blebbing and opening of pannexin 1 (PANX1) channels to allow ATP release into the extracellular space [83]. The secretion of ATP leads to the activation of P2RX7 receptor on DCs, resulting in the promotion of chemotaxis, tumour infiltration by inflammatory cells, and inflammasome-dependent release of the pro-inflammatory cytokines IL-1β and IL-18 [79,92]. Finally, the post-mortem release of HMGB1 is essential to eliciting ICD. HMGB1 is a nuclear non-histone chromatin-binding protein that plays multiple roles [93]. As a nuclear protein, HMGB1 modulates transcriptional activity of various proteins, including p53 and nuclear factor-κB (NF-κB). In the cytosol, it is involved in regulating autophagy [94]. When secreted, HMGB1 can act as a cytokine or as a DAMP [86,95]. In the context of ICD, HMGB1 is secreted at late apoptotic phases or during secondary necrosis [96]. Although a study conducted by Thorburn et al. suggests that some cells undergoing autophagic cell death could induce HMGB1 secretion without losing membrane integrity or succumbing to classical necrosis, the molecular mechanisms that bring about HMGB1 secretion following treatment with ICD inducers have not been fully elucidated [97,98]. When HMGB1 is released from dying tumour cells, protective immunity is stimulated through toll-like receptor (TLR) signalling [79]. Specifically, HMGB1 secreted from dying cells interacts with TLR4 on the surface of DCs, triggering the MyD88-dependent signalling cascade, leading to inhibition of the fusion of phagosomes and lysosomes, ultimately facilitating tumour antigen processing, antigen presentation, and stimulation of the adaptive immune response [87,99]. To date, research has indicated that signal molecules related to multiple cellular processes, such as apoptosis, autophagy, and necrosis, are involved in eliciting ICD. Cellular senescence of tumour cells through p53 activation has also been shown to be associated with increasing tumour immunogenicity through the release of pro-inflammatory cytokines and the recruitment of innate immune cells, such as macrophages and natural killer cells [100,101,102]. The molecular mechanisms underlying ICD are proving to be very complex, and additional insights are warranted to determine the appropriate ICD-inducing treatments, and to better understand how tumour-specific genetic aberrations, intra- and intertumoural heterogeneity, and the TME, impact outcomes of ICD-inducing treatments and other types of immunotherapy.

### 3.2. Radiation-Induced ICD

Radiotherapy and photodynamic therapy (PDT) have been shown to trigger immunogenic cell death (ICD) within tumours. Radiation therapy is used in the management of approximately 50% of all cancer patients [103], and is either administered externally, where a beam of ionising radiation is directed towards the tumour from an external source, or internally (also known as brachytherapy), where radioactive isotopes are implanted within the vicinity of the tumour. The administered radiation causes DNA damage and consequent cell death by directly ionising DNA macromolecules, or by ionising water molecules within the cell. These, in turn, result in the formation of free radicals and reactive oxygen species (ROS) that can react with nuclear DNA. Alternatively, non-ionising radiation can be used to treat tumours by administering a photosensitising agent, such as hypericin, and illuminating the tumour with a specific wavelength of visible light. Any photosensitiser accumulated within the tumour is excited from a ground state, and subsequently releases energy onto nearby oxygen molecules, resulting in the formation of ROS and consequent cellular damage [104]. Although both of these physical treatment modalities target tumour cells by inducing DNA damage, preclinical studies show that immune cell activation is required for an optimal response to treatment. Early work demonstrated that less radiation was needed to control tumour growth in immune competent mice relative to immunosuppressed mice [105], and a later study showed that cytotoxic T-cells enhanced the effect of ablative radiation on local tumour growth [106]. In the latter study, irradiation of primary tumours expressing an antigenic peptide promoted DC maturation and antigen presentation, leading to enhanced antigen-specific T-cell proliferation in tumour-draining lymph nodes [106]. These observations are consistent with the current view that radiation triggers an immunogenic form of cell death that promotes antigen-presenting cell function and activates adaptive antitumour immunity. Radiation-induced cell death exhibits several of the hallmarks of ICD (reviewed in [54,107]), including protection from subsequent tumour challenge following injection of lethally-irradiated cells in mice [108], increased surface expression of calreticulin [109], activation of type I interferon signalling [110], and the secretion of ATP [111] and HMGB1 [112]. Importantly, retrospective analysis of a recently concluded clinical trial in non-small cell lung cancer (NSCLC) shows that radiation synergises with immunotherapy [113], an effect previously observed in preclinical studies [114,115]. While several prospective clinical trials evaluating synergy between radiation and ICD are still underway, studies to date suggest that radiation-induced ICD can prime the tumour to be more responsive to immune-driven therapies, justifying the need to understand and mitigate any drivers of radiation resistance. Radiation-induced ICD, however, seems to be strongly dependent on radiation dose [55]. Radiation doses that are too low may show no effects, since not enough tumour cells are killed to release neoantigens and produce sufficient DAMPs. Radiation doses that are too high may result in no ICD induction, due to direct toxicity on immune cells. Synergy between radiation and ICD may be heavily reliant on accurate and appropriate radiation dosing.

Both tumour cell-intrinsic and extrinsic features promote resistance to radiation therapy. The cell’s intrinsic ability to repair radiation-induced double strand breaks (DSBs) in DNA is key to surviving radiation exposure, and defects in certain DNA repair pathways, such as non-homologous end joining, enhances cellular radiosensitivity [116]. In addition, there is evidence that certain cell subpopulations, such as cancer stem cells (CSCs), may be inherently more radioresistant than other cell types [117], potentially due to an enhanced ability to repair DNA damage or higher intracellular levels of ROS scavengers (discussed in [118]). Cell-extrinsic factors that promote radioresistance include hypoxia, and the radiation responses of local vascular and immune cell populations (reviewed in [118]); thus, both molecular and cellular features of the TME help dictate overall treatment response to radiation. Oxygen, a well-established radiosensitiser, “fixes” DNA damage by reacting with radiation-induced free radicals on the DNA molecule and preventing chemical repair of the lesion [119]. On the contrary, DSBs in the DNA of cells irradiated under hypoxia are easier to repair relative to cells treated in normoxic conditions [120], and clinical studies show that tumour hypoxia is associated with poorer responses to radiation treatment across several cancer types [121,122]. Certain features of the TME, such as hypoxia, render cells radioresistant prior to the onset of treatment; however, radiation-induced changes in the TME can also affect treatment outcomes. As discussed above, radiation promotes antitumour immunity by inducing ICD and, consequently, increasing presentation of tumour antigens; however, radiation can also exacerbate certain immunosuppressive features of the TME. For example, radiation converts latent TGF-β in the extracellular microenvironment to an active form [123], and promotes the recruitment of myeloid-derived suppressor cells (MDSCs) into the tumour bed [124], thereby increasing immunosuppressive signalling within the TME and, ultimately, promoting radiation resistance. Notably, the immune-promoting effects of radiation can be favoured by targeting these suppressive axes with neutralising antibodies [124,125], highlighting the importance of combination therapy to achieve optimal radiation-induced immune priming and tumour control. Again, there are treatment opportunities consisting of combining radiation therapy with liposome-delivered agents that specifically target certain radiation resistance factors within the TME, potentially promoting higher levels of radiation-induced ICD and the consequent development of effective antitumour immunity.

### 3.3. Chemotherapeutic Inducers of ICD

In addition to radiation, certain chemotherapeutic drugs have been observed to induce ICD. Chemotherapeutic ICD inducers are classified as either Type I or Type II, based on the mechanism by which they induce cell death and ER stress [126]. Type I inducers are agents that primarily target cellular processes other than directly inducing ER stress. Examples would include DNA damaging agents, such as the anthracyclines (e.g., doxorubicin, epirubicin), mitoxantrone, belomycin, oxaliplatin, and cyclophosphamide [84,126]. Compounds that target other cellular processes, such as bortezomib, patupilone, and shikonin, have also been identified as Type I inducers [84,126,127,128]. These cytotoxic agents induce cell death in a manner that results in ER stress and the release of ICD-related DAMPs as a secondary effect. Type II ICD inducers are agents that directly target the ER, inducing ICD by either disrupting ER homeostasis or inducing ER stress [86,126]. An example of Type II ICD inducers is hypericin-based photodynamic therapy (Hyp-PDT), where a photosensitiser (hypericin in this case) accumulates and is then activated with light energy. Hypericin localises primarily at the ER and, hence, directly induces ROS generation upon activation, resulting in the induction of ICD that is associated with increased emission of DAMPs relative to Type I inducers [126,129]. Furthermore, the signalling pathways that lead to ICD induction by Hyp-PDT differ from those activated by Type I inducers [126]. Coxsackievirus B3, an oncolytic virus, is another Type II inducer that has been identified as promoting the production of the viral envelope protein that causes ER stress, which leads to cancer cell death [126,130].

As described above, there are few existing chemotherapeutics that are bona fide ICD inducers. Furthermore, these agents typically induce ICD as a collateral effect. While the doses being used are sufficient to bring about cancer cell death, the ICD-related effects may be mild or insufficient to stimulate an adaptive immune response, due to dosage, regional exposure to the candidate drug, and/or other factors, such as a poorly immunogenic TME. Many standard care agents are potent drugs that do not trigger ICD, but do emit some DAMPs that are involved in eliciting ICD. For instance, treatment with either cisplatin or gemcitabine is associated with ATP secretion and HMGB1 release, but not cell surface exposure of CRT [84,131,132]. In recent years, a significant effort has been focused on devising novel combination strategies that would convert non-immunogenic cytotoxic treatments into ICD-inducing regimens. This can be done by complementing existing chemotherapeutic agents with compounds that induce one or more of the missing DAMPs, or by restoring the capacity of poorly immunogenic cancer cells to secrete DAMPs in response to an ICD-inducing treatment [84]. For instance, cisplatin, a non-ICD inducer, has been shown to induce ICD when used in combination with tunicamycin, pyridoxine, or thapsigargin: compounds that induce ER stress [133,134,135]. Similarly, the addition of PX-478, a HIF-1α inhibitor, to gemcitabine, has been shown to induce ICD in pancreatic ductal adenocarcinoma [136]. On the other hand, autophagy-incompetent cancer cells fail to induce ICD when exposed to anthracyclines, due to their inability to secrete large amounts of ATP [137]. In this case, the administration of 6-*N*,*N*-diethyl-d-*β*-*γ*-dibromomethylene adenosine triphosphate (ARL67156), an extracellular nucleotidase inhibitor, was able to restore ATP secretion and anthracycline-induced ICD in these cells [137]. This strategy could also be applied to combination treatments comprising of chemotherapeutics that fail to promote ATP secretion. Similarly, some studies have suggested that HMGB1-deficient cells could have their immunogenicity restored through provision of an exogenous TLR4 agonist, such as dendrophilin [84,99]. All of these studies point to the importance of comprehensively understanding the mechanisms of actions of drugs when used in combination, the genetic aberrations and immunogenicity of the cancer cells, as well as the TME, in order to determine the optimal ICD-inducing treatments for each patient.

## 4. Nanomedicines to Modulate the TME and ICD

Chemotherapeutics are an invaluable component of many cancer treatment regimes. Unfortunately, treatments that are used clinically are small molecules that may be therapeutically limited by (i) poor aqueous solubility, (ii) rapid depletion from circulation via metabolic and elimination pathways or by poor adsorption, and/or (iii) suboptimal biodistribution properties, which result in significant toxicities and/or the inability of the active agent to reach therapeutically relevant concentrations at the tumour site, at the right time and in the right concentration. By virtue of direct conjugation to change the physical properties of the molecule (e.g., albumin-conjugated paclitaxel [138,139]) or physical protection from the in vivo environment using a hydrophilic shell (e.g., liposomes, micelles, dendrimers), nanocarriers have provided a means of facilitating the in vivo delivery of poorly water-soluble drugs, and a method of manipulating the pharmacokinetics, biodistribution, and stability of associated drug molecules. Further, nanomedicines have shown promise as a strategy to facilitate the effective delivery of TME-targeting therapeutics to sites of tumour growth [140].

### 4.1. Liposomes

**Figure 2:**
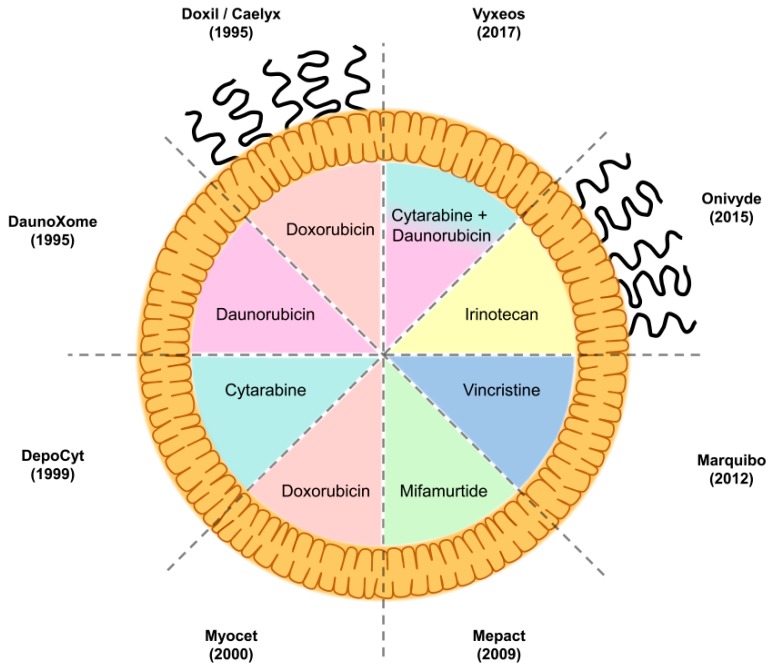
Liposomal formulations used in the clinic. Commercial name and year of first approval by the authorities is provided for each of the formulations. Loaded drug and decoration of liposomal surface with PEG (in the case of Doxil™/Caelyx™ and Onivyde™) is represented in the schematics. Doxil™/Caelyx™ was the first formulation to receive approval in 1995, and consisted of PEGylated liposomal doxorubicin. Vyxeos™, approved in 2017, is, so far, the last approved liposomal formulation, and it contains a combination of two encapsulated drugs at a fixed 5:1 molar ratio of cytarabine/daunorubicin. Patisiran, a small interfering RNA (siRNA) formulation for treatment of hereditary amyloid transthyretin (ATTR) amyloidosis, will likely be approved in 2018, the year that this review was written.

Of the various nanoformulations available, liposomes have emerged as the most clinically successful, in terms of the number of formulations approved (Figure 2), and are actively used as mainstay treatments in oncology [141]. Liposomal formulations have been approved for the treatment of several cancer modalities (Table 2). This has arisen through a combination of factors, such as ease of production, reproducibility, cost, and amenability to manufacturing scale-up and sterile preparation.

Generally formed from a combination of phospholipids and cholesterol, liposomes are bilayered vesicles in the size range of 50–500 nm. While lipophilic drugs can be incorporated into the lipid membrane, most liposomes contain the majority of their payload encapsulated within the aqueous interior. Drug molecules can be loaded into liposomes passively, via addition during liposome synthesis; this was used as a method to trap cytarabine in Vyxeos™. More commonly, drugs are actively loaded into preformed liposomes using a process that is referred to as remote loading. Drugs cross the lipid bilayer down the drug concentration gradient, then are trapped within the liposome via ionisation as a result of a high or (more commonly) low pH interior environment [142], or via complexation, e.g., to metal ions [143]. The physicochemical change that the drug molecule undergoes within the liposome regenerates the concentration gradient of the drug, driving further loading. As such, drug loading into liposomes can be highly efficient, often resulting in entrapped drug concentrations much higher than the solubility of the drug, and with a greater than 500-fold improvement in apparent drug solubility.

A further advantage to the use of liposomes as anticancer drug delivery vehicles is that they provide opportunities for surface functionalisation. The circulation half-life of liposomes can be further prolonged through the incorporation of polyethylene glycol (PEG) chains into the lipid bilayer [144]. The PEG polymers form a protective corona that prevents surface–surface aggregation [145], and delays recognition and engulfment by the mononuclear phagocyte system (MPS) [146,147]. However, on repeated administration of PEGylated liposomes, an accelerated clearance from the blood is observed, termed the accelerated blood clearance (ABC) phenomenon [148]. This is a result of an anti-PEG IgM response produced from a first exposure to PEG liposomes, and the subsequent activation of the complement system, excessive liposomal opsonisation, and liver uptake of liposomes with subsequent doses [149,150]. Perhaps as a result of this mechanism, only two liposome formulations currently approved to treat cancer are PEGylated (Doxil™ and Onivyde™), but this effect may also be species specific, or may be prevented by encapsulation of certain drugs/drug candidates.

Liposomal encapsulation has eliminated the need of toxic excipients in the formulation of poorly water-soluble compounds, such as paclitaxel [151], and altered the systemic exposure and biodistribution of compounds, such as doxorubicin [152,153] and vincristine [154], to limit systemic drug-induced toxicities. Liposomal drug delivery is further utilised to skew the pharmacokinetics and biodistribution of therapeutics, so as to prolong and enhance the exposure of drug molecules to tumours themselves. The capacity to overcome formulation and toxicity difficulties, as well as to enhance drug candidate distribution to sites of tumour growth, makes liposomes ideal candidates as a formulation strategy for agents designed to modulate the TME.

### 4.2. Passive Targeting and the EPR Effect

**Figure 3:**
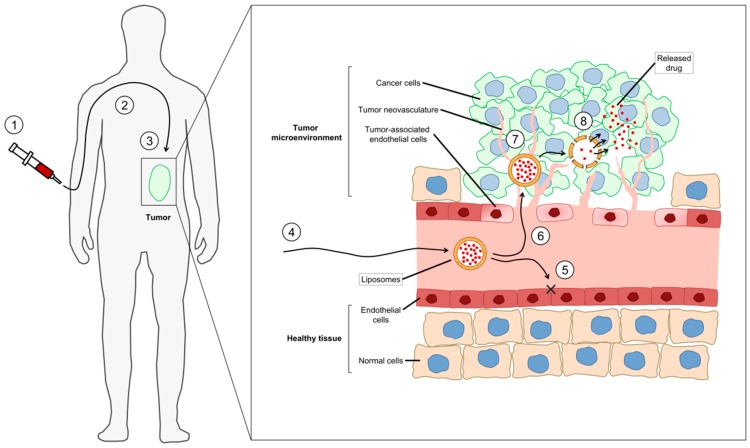
Passive targeting of liposomal formulations by the enhanced permeability and retention (EPR) effect. (**1**) Liposomes are administered intravenously to cancer patients. (**2**) They enter the blood flow and remain in circulation for extended periods of time, due to their large particle size and improved pharmacokinetics, while the drug cargo is protected from degradation. (**3**, **4**) Liposomes arrive at the tumour through the blood vessels. (**5**) They are not able to extravasate to healthy tissue because of the compact endothelial cell layer (×) that forms the capillaries. (**6**) However, they escape from blood circulation through the enhanced permeability of the tumour neovasculature, which is poorly formed, inflamed and “leaky”. (**7**) Liposomes are retained in the tumour microenvironment, since the associated lymphatic vessels are impaired. (**8**) The encapsulated drug is released from the accumulated liposomes into the tumour microenvironment and finally internalised by cancer cells. The arrows show the distribution of the liposomal drug from the intravenous administration to the accumulation and release into the tumour microenvironment.

First termed by Maeda in the 1980s [155], the enhanced permeation and retention (EPR) effect has been consistently cited as the primary mechanism whereby nanoparticle-associated drug molecules can enhance drug levels in solid tumours when compared to the drug given in a non-nanomedicine formulation. There is a reasonable amount of controversy about the importance of the EPR effect, including (i) heterogeneity of the effect, (ii) the presence of the nanoformulation of the drug at the tumour does not necessarily mean that the drug is available to interact with target cells, (iii) the enhanced circulation lifetime may contribute more to efficacy, and (iv) the effect may be very model dependent. Recent reports have highlighted the caution that should be taken in contributing this tumour drug-accumulation phenomenon entirely to the EPR mechanism [156,157,158,159]. Regardless, the EPR effect (Figure 3) is the cumulative result of a combination of physiological properties unique to the TME, in combination with the pharmacokinetic attributes of nanoparticles, which will include how well the associated drug remains associated after administration. The three general components of EPR effect are:

(1) Nanoparticle pharmacokinetics. Small molecule chemotherapeutic drugs and peptides generally have short in vivo half-lives as a result of poor physical and metabolic stabilities coupled with rapid renal and hepatic eliminations. Association of a drug molecule with a nanoparticle, however, confers to it the properties of the nanoparticle system which are designed to be retained in the plasma compartment for extended time periods following intravenous administration. The relatively large nanoparticle (10 nm to 200 nm) and its associated corona (particle with bound proteins) can often-times shield the associated drug from the in vivo environment. As such, while the drug is still associated with the nanoparticle, it is often still in an active form, and is available for therapeutic activity once dissociated from the nanoparticle.

(2) Tumour vasculature. The rapid growth of solid tumours requires the delivery of extra nutrients that the body’s normal vasculature cannot provide. As such, tumours orchestrate the formation of their own system of blood vessels in a process known as angiogenesis (see above). The rapid propagation of tumour-associated blood vessels results in chaotic and poorly formed “leaky” vessels. The long circulation time of nanoparticles means that they have multiple exposures to the tumour vasculature, and they can extravasate from the blood through leaky vessels into the TME over time.

(3) Impaired lymphatic drainage. In normal physiology, the lymphatic network of vessels returns fluid and large molecules that have extravasated into tissue, back into the systemic circulation. The high interstitial pressure of the TME, in conjunction with rapidly growing cells, puts increased pressure on tumour lymphatic vessels, impairing their functionality. The presence of lymphatic vasculature within solid tumours is variable, and those found in the TME are significantly impaired [160]. Extravasated nanoparticles therefore remain in the TME, and are available for uptake into tumour cells, or to release their payload in the TME.

### 4.3. Liposomal Drugs Normalising the Tumour Neovasculature

Chemotherapy generally follows a treatment schedule designed to deliver a high dose of drug, and a recovery period prior to subsequent doses to allow healthy cells/tissues to recover. However, it has also been suggested that within this recovery period, cancer and stromal cells, and specifically vascular endothelial cells, also have the opportunity to recover [161]. “Metronomic dosing” has been proposed as alternative dosing schedule, where submaximal dosages are administered via long infusions, and there is a shorter recovery interval between treatments. As such, a low concentration of drug is systemically available over long periods of time, instead of a large amount being available for a relatively limited timeframe. Various chemotherapeutic treatments have the capacity to kill tumour-associated vascular endothelial cells, and newly formed angiogenic cells are particularly susceptible to metronomic dosing [40,41]. Metronomic dosing has been shown to kill more cells in angiogenic blood vessels, producing a vascular normalisation effect that can improve the blood flow to tumours, reduce tumour hypoxia, and increase apoptotic effects following treatment. Perhaps most importantly, vascular normalisation has been associated with improved delivery of small molecule drugs, suggesting that metronomic dosing could be used in combination with small molecules given at high doses, provided the sequencing of the drug combination is correct. Mirroring the systemic drug availability of metronomic dosing, liposomes have the capability to alter the pharmacokinetics of small molecule chemotherapeutics, prolonging the systemic circulation time of a chemotherapeutic payload significantly, while minimising peak drug concentrations always seen when using high-dose strategies. Slow release of a chemotherapeutic drug from a liposome over time in the circulation provides a continuous source of the therapeutic to a tumour over time, effectively behaving as a low concentration infusion. As such, liposomal delivery of chemotherapeutics has been shown to facilitate the normalisation of tumour vasculature, improving drug penetration and anticancer efficacy, as exemplified below. The liposomal formulations modulating the TME are summarised in Table 3, and their effects on the TME are described below.

#### 4.3.1. Liposomal Doxorubicin

Liposomal doxorubicin was the first of many liposomal anthracycline formulations approved for the clinical treatment of cancer. It was interesting that Doxil™ was first approved for AIDS-related Kaposi’s sarcoma, which is an endothelium-related disease, yet the antiangiogenic activity of encapsulated doxorubicin was only recently reported. Initial studies in murine orthotopic models of glioblastoma indicated, via MRI imaging, the incidence of haemorrhaging within the tumours of mice administered multiple doses of PEGylated liposomal doxorubicin, whereas no haemorrhaging was observed in mice administered saline or free doxorubicin [162]. While the mechanism was not elucidated, it was hypothesised that the decrease in vascular function was either the direct result of vascular cell death via the accumulation of doxorubicin within the TME [163], or a secondary effect arising from the reduction in VEGF with the death of tumour cells.

Another interesting example concerns tumours initiated following injection of a P-glycoprotein (Pgp)-upregulated colorectal cancer cell line. These cells showed resistance to doxorubicin in vitro; however, the same cells grown in vivo demonstrated comparable growth delay patterns to the wild-type cell line following the administration of liposomal doxorubicin. The discrepancy between in vitro and in vivo results was shown to be at least partly caused by increased vascular cell death in mice bearing the doxorubicin-resistant cell line when compared to the wild-type tumours. It was suggested that the liposomal doxorubicin facilitated the death of vascular endothelial cells. This, in turn, reduced the growth rate of the tumour in vivo, demonstrating the importance of vascular cell death in tumour growth [164].

#### 4.3.2. Liposomal Irinotecan

Tumour normalisation effects have been reported following administration of liposomal irinotecan. A liposomal formulation of irinotecan (Irinophore C™) decreased both the density of tumour cells and tumour vasculature (CD31^+^) cells in subcutaneous colorectal tumours of mice treated weekly for 6 weeks, when compared to saline-treated mice [165]. Irinophore C™ treatment further resulted in a greater vascular density per tumour region when compared to saline-treated controls, resulting in less hypoxia when compared to tumours from animals treated with saline. Following identification of the higher vascular density, the authors investigated whether pretreatment with Irinophore C™ would improve the tumour penetration of a subsequent treatment. Both 5-fluorouracil (5-FU) and doxorubicin had greater distribution throughout the tumours in mice pretreated with Irinophore C™ than in mice pretreated with saline. This was particularly pronounced when the accumulation of drug (doxorubicin) was normalised to tumour tissue density, where a greater than 2-fold increase in drug accumulation was observed in comparison to the control group. A screen investigating the expression of angiogenic factors indicated that when compared to saline-treated controls, the proangiogenic VEGF and IL8 were downregulated, and the angiogenic suppressor TIMP-1 was upregulated in Irinophore C™-treated mice [165].

A separate study revealed that weekly injections of liposomal irinotecan (Irinophore C™), liposomal vincristine, or liposomal doxorubicin (Doxil™) all reduced the growth of a subcutaneous murine model of glioblastoma, and increased the percentage of perfused tissue per tumour when compared to the saline control [41]. Further, the density of functional blood vessels was increased in the Doxil™-treated group when compared to the saline-treated group. Perhaps surprisingly, in an orthotopic model of the same cell line, tumour perfusion was decreased in all treated groups compared to the saline group, as assessed by Hoechst 33342 staining. As Hoechst is a P-glycoprotein (pgp) substrate, and does not cross the blood–brain barrier (BBB), the authors postulated that vascular normalisation may have led to restoration of the normal BBB.

**Figure 4:**
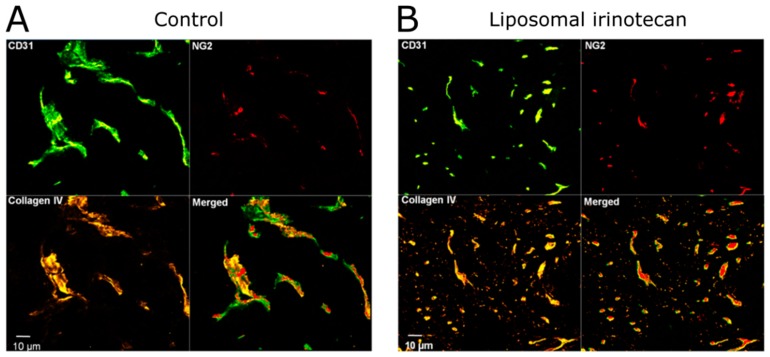
Normalisation of tumour neovasculature by liposomal irinotecan in a mouse model of glioblastoma. After treatment with liposomal irinotecan (Irinophore C™) once weekly for 3 weeks, Verreault et al. recognised a significant reduction of the diameters of tumour blood vessels (identified by the CD31 marker, in green) in the subcutaneous glioblastoma xenograft. In contrast, the number of pericytes (NG2, in red) and the basement membrane coverage of blood vessels (collagen IV, in yellow) increased after the therapy. Taking the data together, fewer immature tumour vessels (CD31^+^, NG2^−^, and collagen IV^−^) were present in the TME after administration of liposomal irinotecan. Adapted with permission from [41]. Copyright 2011 by Verreault et al.

It was shown that irinotecan, doxorubicin, and vincristine are all potent against vascular cells in culture, when under proliferative conditions versus non-proliferative conditions. As such, if these drugs are released from liposomes in the TME, it is possible that they can act on proliferating (i.e., angiogenic) blood vessels (Figure 4). Based on this evidence for tumour vessel normalisation, the capillary permeability in orthotopic tumours was measured in mice treated with Irinophore C™, and compared to that in mice treated with saline, where a significant decrease in capillary permeability in Irinophore C™-treated mice was observed [41].

Additional studies sought to assess the efficacy of 5-FU administered following treatment and vasculature normalisation with Irinophore C™. Irinophore C™ treatment lead to the initial decrease in vascular function; however, after 3 rounds of chemotherapeutic treatment, the percentage of vascular cells in tumours were significantly increased when compared with saline-treated control mice [166]. Irinophore C™ and 5-FU could not be given concurrently, due to excessive toxicities, and so 5-FU was administered 7 days following the final dose of Irinophore C™. The amount of 5-FU in the excised tumour was significantly increased in mice pretreated with Irinophore C™ instead of saline, and this difference increased with every additional dose of Irinophore C™ administered prior to 5-FU administration. These changes resulted in improved efficacy in the treatment of subcutaneous colorectal adenocarcinoma tumours (HT-29 cells) in a mouse model. Mice treated with irinotecan, sequentially followed by 5-FU treatment, had delayed tumour growth when compared to mice administered with Irinophore C™ or 5-FU alone [166]. These studies demonstrate the potential for liposomal formulations of chemotherapeutics to be used not only for direct activity against tumours, but for their vasculature-normalisation capabilities, an effect believed to be on tumour-associated vascular endothelial cells.

#### 4.3.3. Liposomal Paclitaxel

While not yet approved in American or European countries, a liposomal paclitaxel formulation has been approved for the treatment of ovarian cancer, breast cancer, and non-small cell lung cancer in China. In a series of studies, the Dellian group demonstrated the antivascular effects of paclitaxel when delivered in a cationic liposomal formulation. Initial studies demonstrated that, although this liposomal formulation of paclitaxel showed comparable in vitro efficacy with Cremophor^®^-solubilised paclitaxel against a human melanoma cell line, it had an improved in vivo activity against a humanised model of melanoma in SCID mice. Further, the liposomal formulation, but not the Cremophor^®^ formulation, reduced tumour vessel density in this in vivo model [167]. The group further confirmed the antivascular effects of liposomal paclitaxel in a hamster model of subcutaneous melanoma. Intravital microscopy revealed a decrease in tumour-associated functional vessel density after treatment with liposomal paclitaxel of up to 50%, when compared to paclitaxel alone (solubilised in cremaphor). Further, following treatment with liposomal paclitaxel, the increase in tumour vessel density associated with increased tumour mass that was seen in control-treated animals was not observed. Together, these physiological changes resulted in tumours with significantly less blood perfusion [168]. Contributing to a decreased tumour perfusion, both empty and paclitaxel-containing liposomes induced platelet adhesion to tumoural microvessel walls, and paclitaxel liposomes produced microthromboses that occluded microvessels after treatment for 3 consecutive days [169]. Further, liposomal paclitaxel pretreatment increased the leakiness of tumour-associated microvessels, such that after a 15 min infusion, a greater amount of fluorescently-tagged albumin was present in the tumour’s extravascular space when compared to tumours previously treated with paclitaxel or empty liposomes [170].

Taken together, these studies demonstrate the capacity of paclitaxel to directly interact not only with tumour cells, but also alter the tumour vasculature. While liposomal paclitaxel had greater efficacy in treating subcutaneous melanoma tumours when compared to results in animals treated with Cremophor^®^-solubilised paclitaxel, the changes in tumour growth rates achieved with the liposomal formulation could be attributed to multiple tumour and TME factors. The group briefly examined co-administration of unencapsulated cisplatin with the paclitaxel liposomes, and they demonstrated a further improvement in in vivo efficacy [170], however, it was not clear whether the combined effect was the result of complementary molecular mechanisms, or was facilitated by changes in the tumour’s vascular system that had been compromised by liposomal paclitaxel.

### 4.4. Liposomal Formulations Affecting Immune Cells in the TME

The slow release of drug(s) from liposomes mimicking metronomic dosing will also modulate the behaviour and content of immune cells present in the TME, although this has yet to be optimised. Ideally, a nanomedicine should have an enhanced inhibitory effect on immunosuppressor cells (regulatory T-cells, M2-polarised tumour-associated macrophages, myeloid-derived suppressor cells) and engender higher activation of immune cells that promote antitumour effects (CD8^+^ T-cells, NK cells). It is important to recognise that liposomes, like all nanomedicines, are known to be eliminated, in part, by cells of the mononuclear phagocyte system (MPS); mainly monocytes and macrophages [171]. The process involves, first, interaction of liposomes with serum proteins which effectively opsonise them, marking them for elimination by MPS cells. In the case of nanomedicines with associated cytotoxic drugs, this process may cause depletion of phagocytic cells as a direct cytotoxic effect of the drug. While this is an effect on immune cells, it is not restricted to the TME but occurs in organs of the MPS, such as the liver, lymph nodes, and spleen. Furthermore, while this effect has been generally associated with immunosuppression, it does not positively influence the balance between M1 and M2-polarised tumour-associated macrophages in the TME. To our knowledge, only two liposomal formulations have been characterised for having an actual beneficial impact on immune cells in the TME: liposomal doxorubicin and liposomal mifamurtide. The effects of these two liposomal formulations are summarised below. It is important to note that other liposomal formulations may enhance immune responses, but only mifamurtide was specifically designed for this activity.

#### 4.4.1. Liposomal Doxorubicin

A recent study evaluated the ability of liposomal doxorubicin (Doxil™) to boost the antitumour response of different cancer immunotherapies [172]. First of all, liposomal doxorubicin presented therapeutic benefits when tumours were grown in immunocompetent but not in immunocompromised mice, demonstrating that the presence of an intact immune system is required for the activity of the liposomal formulation. Pharmacodynamic studies in mice provided evidence that the amount of tumour-infiltrating T_reg_ cells decrease upon administration of liposomal doxorubicin. Furthermore, the quantity of CD8^+^ T-cells in the TME was augmented when liposomal doxorubicin was given in combination with anti-PD-L1 antibodies [172]. Liposomal doxorubicin also synergised with anti-PD-1 and anti-CTLA-4 antibodies. Treatment with liposomal doxorubicin enhanced the expression of CD80 on mature dendritic cells, and monocytic and granulocytic myeloid cells, and this costimulatory phenotype may be related and facilitate the activation of the associated antitumour CD8^+^ T-cell response.

The effects of liposomal doxorubicin were also studied on tumour-associated macrophages [173]. Liposomal doxorubicin (both PEGylated and non-PEGylated formulations) successfully increased the concentration of drug in the tumour tissue 4-fold, in comparison to free drug. However, the visualisation of doxorubicin fluorescence poorly correlated with CD11b^+^ tumour-associated macrophages, implying that the increased accumulation of drug in the tumour was not related to the uptake of liposomal drug by macrophages, but to alternative mechanisms known to enhance tumour vascular permeability [173]. Another study investigated in further detail the role of tumour-associated macrophages in the antitumour activity of Doxil™. In this case, by evaluating the therapeutic activity of Doxil™ in B16.F10 melanoma-bearing mice in the presence or absence of macrophages (suppressed by liposomal clodronate), the results again attributed direct cytotoxicity as the main therapeutic mechanism, but pointed out partial contributions to the inhibition of angiogenesis mediated by tumour-associated macrophages [174]. However, increased immunogenicity of surviving tumour cells after treatment with Doxil™ may be connected to the upregulation and surface expression of MHC-I and Fas. These changes, in turn, will lead to a sensitisation of tumour cells to CD8^+^ T-cells and Fas-mediated death, as demonstrated in vitro [175]. In the same study, the combination of the immunostimulatory cytokine IL-18 and liposomal doxorubicin significantly suppressed tumour progression in vivo, relative to the use of single agents. Another study analysed modulation of the immune system while treating breast cancer with temperature-sensitive liposomal doxorubicin and locally-administered CpG as immune adjuvant [176]. The combined treatment resulted in increased CD4^+^ and CD8^+^ T-cells, and reduced myeloid-derived suppressor cells not only in the treated tumour, but also in the contralateral tumour site. All these observations link use of liposomal doxorubicin to immune mediated effects on the tumour.

#### 4.4.2. Liposomal Mifamurtide

Mifamurtide is muramyl tripeptide phosphatidylethanolamine (MTP-PE), a lipophilic synthetic derivative of a peptide naturally occurring in the bacterial wall of the bacteria Bacillus Calmette–Guerin. Mifamurtide, associated with liposomes, administered to osteosarcoma patients in combination with chemotherapy, had improved survival in comparison to patients that only received chemotherapy [177]. In contrast to cytotoxic drugs used in conventional chemotherapy, mifamurtide does not attempt to directly kill tumour cells but, instead, stimulates monocytes and macrophages to release proinflammatory cytokines, including TNF-α, IL-1, IL-6, IL-8, nitric oxide, prostaglandin E2, and prostaglandin D2 [178]. Liposomal mifamurtide also induces the expression of adhesion molecules such as lymphocyte function-associated antigen 1 (LFA-1), intracellular adhesion molecule 1 (ICAM-1), and human leukocyte antigen DR (HLA-DR) [178]. Further, a recent study investigating the capacity of tumour-associated macrophages to cause direct antitumour activity against osteosarcoma cells after activation by liposomal mifamurtide highlighted the importance of the immune system [179]. In a series of in vitro experiments, the authors polarised monocytes into M1-like (classically activated) and M2-like (alternatively regulated or immune suppressors) macrophages, and co-incubated them with osteosarcoma cells. On the one hand, stimulation of M1-like macrophages with liposomal mifamurtide inhibited growth of tumour cells, but this seemed to occur only in the presence of interferon-γ. On the other hand, activated M2-like macrophages exhibited low antitumour activity, and growth of tumour cells was only reduced when the anti-EGFR antibody cetuximab was co-administered to cancer cells in a process involving antibody-dependent cell-mediated phagocytosis [179]. The main role of liposomal mifamurtide may be inducing the expression of proinflammatory cytokines that lead to an M1 macrophage response [180].

### 4.5. Liposomal Formulations to Enhance Radiation-Induced ICD

Few studies have directly assessed the ability of liposome-delivered agents to synergise with radiation and promote ICD. One strategy reported by Chamoto et al., used liposomes to co-encapsulate a tumour-specific peptide (OVA) and the TLR9 agonist CpG, a commonly used vaccine adjuvant [181]. Intradermal injection of the CpG + OVA liposome near the tumour-draining lymph node alone was not able to eradicate OVA-expressing Lewis lung carcinoma; however, irradiating the tumours with two 14 Gy fractions prior to administering the liposomes cured 60% of the treated mice. Although both radiation and liposome treatment alone induced some level of OVA-specific cytotoxic T lymphocytes (CTL), the combination increased this population by nearly two-fold relative to either monotherapy. In addition, irradiating the tumour promoted the infiltration of CD11c^+^ cells, tentatively suggesting that radiation-induced enhancement of antigen-presentation was promoting a synergistic response with the liposome-based tumour vaccine [181]. Importantly, co-administration of CpG and OVA, rather than either component alone, was required for a synergistic expansion of tumour-specific CTL with radiation [181]. In addition, the same group had previously reported that liposome-based immune adjuvants promoted a stronger immune response than administration of the adjuvants in their soluble form [182,183], highlighting the value of liposomal delivery and co-encapsulation of immune-promoting agents to control tumour growth.

Other strategies to enhance radiation-induced ICD involve targeting features of the TME that limit radiation therapy’s cytotoxicity, such as hypoxia [184]. In some cases, researchers have developed hypoxia-sensitive nanoparticles to direct the release of cytotoxic chemotherapy within this specific microenvironment. Recently, Liu et al. developed hypoxia-activated liposomes that could synergise with radiation therapy to treat a preclinical model of glioma [185]. These liposomes used lipid molecules conjugated to metronidazole (MI), a type of nitroimidazole. This class of organic compounds is commonly used when developing hypoxia-responsive agents, since they are reduced from a hydrophobic compound to a hydrophilic aminoimidazole under hypoxic conditions. Thus, liposomes constructed from MI-conjugated lipids destabilised under hypoxia, releasing their contents specifically within oxygen-poor areas of the tumour [185]. The researchers used the hypoxia-activated liposomes to deliver doxorubicin to the tumour, which synergised with radiation to inhibit both intracranial and subcutaneous glioma tumour growth [185]. In addition to delivering chemotherapy to the radioresistant hypoxic regions of the tumour, the structural components of the MI-containing liposomes, themselves, could help sensitise hypoxic cells to radiation, since the released MI acts as an oxygen mimetic that can cause radiation-induced DNA damage. Another strategy for alleviating hypoxia-induced radioresistance is to increase the levels of oxygen, or oxygen-like compounds within the tumour. Zhang et al. used liposomes to deliver antioxidant enzymes to the tumour, with the intent of catalysing reactions that would increase intratumour oxygen levels [186]. Using phospholipids conjugated to a cisplatin prodrug (Pt (IV)), the researchers loaded Pt (IV)-liposomes with catalase, which decomposed hydrogen peroxide to molecular oxygen in vitro [186]. The catalase-loaded Pt (IV)-liposomes were able to sensitise hypoxic (1% O_2_) tumour cells to ionising radiation in vitro, and the authors provided some evidence that these liposomes relieved tumour hypoxia in vivo, whereas catalase-free Pt (IV)-liposomes had no effect on tumour hypoxia [186]. Ultimately, catalase-loaded Pt (IV)-liposomes synergised with radiation to control subcutaneous tumour growth in mice, though alone, these liposomes failed to inhibit tumour growth despite including the cisplatin prodrug. Another group used liposomes to deliver oxygen-carrying perfluorohexanes to the tumour, resulting in a modest, but significant, enhancement of radiation-induced control of tumour growth [187]. The studies reviewed illustrate that liposomes can be engineered to release their contents specifically within hypoxic regions of the tumour, and direct additional cytotoxic agents to these inherently radioresistant tissues. In addition, liposomes can be used to deliver molecules or enzymes that enhance oxygen availability within the tumour, thereby reducing levels of tumour hypoxia and promoting subsequent responses to radiation treatment. Of note, preclinical studies show that radiation itself enhances liposome uptake and distribution within the tumour [188,189], an effect that may contribute to observed synergy between radiation therapy and liposomal nanomedicines.

### 4.6. Liposomal Formulations to Enhance Chemotherapy-Induced ICD

The success of nanomedicinces, in particular, liposomal drugs and drug combinations, has been discussed above. The advantage of these formulations likely involves their ability to alter the pharmacokinetic profile and biodistribution of the associated drug(s). As a consequence of this treatment, efficacy can be enhanced and the formulations can exhibit reduced toxicity. At the time that this review is written (2018), the use of nanoparticles to induce ICD has not been thoroughly explored. Nanoparticles are known to interact with the immune system and can be engineered to attain the desired immunomodulatory effects [190,191]. It will be most interesting to determine whether or not the use of purposefully designed nanoparticles, particularly liposomes, would potentiate the immune-stimulatory effects of chemotherapeutic ICD-inducing treatments. In a recent study, Zhao et al. have demonstrated that encapsulation of oxaliplatin and doxorubicin in polymer-based mPEG-PLGA nanoparticles resulted in formulations that induced potent ICD effects, characterised by increased secretion of DAMPs, increased tumour infiltrating cytotoxic T-cells, and enhanced therapeutic efficacy in immunocompetent models [192]. The improved accumulation of ICD inducers at the tumour site could lead to increased ICD-associated signalling and more potent antitumour immune responses. The authors have also shown that encapsulation of non-ICD inducers, such as 5-FU and gemcitabine, did not bring about ICD induction, suggesting that the use of nanoparticles is a formulation strategy rather than a means of eliciting ICD with non-ICD inducers. In another study, Rios-Doria et al. demonstrated that Doxil™ (liposomal doxorubicin) but not doxorubicin, has potent antitumour activity against established tumours in immunocompetent mice [172]. The authors found that the liposomal formulation was able to reduce tumour-infiltrating regulatory T-cells, increase cytotoxic T-cell infiltration, and increase the expression of CD80 on mature DCs. Similarly, Huang et al. demonstrated that ultrasound-controlled release of doxorubicin from liposome–microbubble complexes was associated with more potent ICD effects [193]. While current data suggest that improved delivery of chemotherapeutic ICD inducers may augment the immunogenicity of ICD treatments, it would be important to understand how different types of nanoparticles and their immunogenicity potential would impact the effectiveness of ICD-inducing treatments. For example, liposomes are known to be engulfed by macrophages, and are generally considered to be poorly immunogenic or even immunosuppressive [194]. Studies completed in our laboratory in the past have shown increased tumour accumulation of doxorubicin using two different liposomal formulations relative to free drug, and this increase in tumour drug concentration was not associated with uptake of liposomal doxorubicin by tumour-associated macrophages [173]. However, a study completed recently by another group has shown that treatment with PEGylated liposomes leads to decreased interferon-γ production by tumour-associated macrophages [194]. Whether or not the use of liposomes with different size, charge, and lipid compositions would affect the immunogenicity of chemotherapeutic ICD treatments remains to be explored.

## 5. Future Directions

Liposomal formulations that are currently used in the clinic were reported to reduce the formation of tumour neovasculature in the TME, as illustrated by studies with liposomal doxorubicin, liposomal irinotecan, and liposomal paclitaxel. However, the research investigating the effects of liposomes on the tumour vasculature is still scarce. It should be noted that some drugs have been described as inhibitors of tumour neovasculature (e.g., daunorubicin [195] and vincristine [196]) and they have been encapsulated in liposomal formulations approved for clinical use. Therefore, it would be most interesting to investigate whether the corresponding liposomal formulations of such compounds, namely DaunoXome™ for daunorubicin and Marquibo™ for vincristine, exhibit, or even enhance, antiangiogenic properties. At a preclinical level, the encapsulation of antiangiogenic drugs, such as sunitinib and sorafenib, has been attempted, in order to increase their activity. Sunitinib liposomes were tested, in combination with liposomal irinotecan against a PC12 neuroendocrine tumour [197] and in combination with vinorelbine liposomes, for the treatment of invasive breast cancer [198]. Sorafenib was co-encapsulated in liposomes with photocyanine [199], gadolinium [200], and siRNA [201]. Further studies with these formulations may clarify if such strategies have the potential to enhance suppression of tumour neovasculature.

Therapeutic antibodies acting against angiogenesis were also coupled to liposomes. Researchers prepared bevacizumab–liposome conjugates to prolong the residency of bevacizumab in the vitreous after intravitreal administration [202]. In another study, bevacizumab was conjugated to cationic liposomes to enhance the targeting potential to the tumour vasculature [203]. Similarly, one can envisage therapeutic antibody–liposome conjugates as a way to modify the biodistribution of the therapeutic antibodies, increase their concentration in the TME, and enhance their antiangiogenic activity. For example, enhancement of direct apoptosis was observed for a multivalent liposomal formulation of rituximab [204]. Further research should tell if direct effects through cell signalling are enhanced in the case of liposomal formulations of anti-VEGFR2 antibodies. Liposomes were also targeted to the tumour vascular endothelial cells through peptides such as RGD [205] and NGR [206], antibodies [207] and antibody fragments [208]. Targeting of liposomes to specific cell subtypes is conceptually a promising strategy, and it will be undoubtedly further investigated in the future. If successful, significant drug development research is required if any such targeted formulation ever turn into clinically approved agents.

Clinical liposomal formulations were also studied for their positive modulation on the immune cells present in the TME. Liposomal doxorubicin and liposomal mifamurtide enhanced the host immune response in different fashions. Do liposomal cytarabine, daunorubicin, vincristine, and irinotecan have an effect on the immune components of the TME? Liposomes have also been targeted to macrophages by several mechanisms, including peptides, antibodies, and lectins [209]. In preclinical studies, complement C3-mediated targeting of liposomes to myeloid-derived suppressor cells suggested that this could be an important tool for restoring antitumour activity [210]. In the context of developing liposomes for ICD induction, first, it would be of interest to evaluate whether the improved efficacy of already approved liposomal formulations is related to enhanced antitumour immune responses. Additionally, certain scientific questions must be addressed. For example, how do the different physiochemical properties of nanoparticles affect the efficacy of ICD-inducing treatments? It is evident in the literature that the use of nanoparticles may enhance the antitumour immune response, and understanding the precise mechanisms of actions associated with different types and features of nanoparticles would be crucial to designing ICD-inducing nanomedicines. It would also be of interest to determine if the combined use of nanoparticles and existing chemotherapeutic, targeted agents, and immunotherapy is more effective than relying on a single ICD-inducing compound. Furthermore, liposomes are reported to synergise with radiation and determining the specific contribution of ICD to this enhancement of therapeutic efficacy may be crucial for the development of new combinatory strategies. We are at the beginning of a new and very promising phase of immunotherapy development.

## 6. Conclusions

Liposomes are a tool to deliver molecules to regions of cancer growth, either through slow release of drug from liposomes in the blood, or release of liposomes that have accumulated in tumours via the EPR effect. Liposomal formulations of drugs have translated into increased efficacy in killing tumour cells, but also affecting different tumour-promoting cell subtypes in the TME, such as vascular endothelial cells, regulatory T-cells, and other cells from the myeloid linage. Three liposomal formulations used to treat cancer in humans were reported to inhibit angiogenesis in the TME: liposomal doxorubicin, liposomal irinotecan, and liposomal paclitaxel. Furthermore, two approved liposomal formulations were reported to enhance tumour-associated immune responses: liposomal doxorubicin and liposomal mifamurtide. Even though research involving liposomes and ICD is still limited, we believe that liposomes are best suited to enhance radiation and chemotherapy-induced ICD. In the latter case, research efforts should be put, first, at identifying drug combinations effective at triggering immunogenic death of tumour cells. At a later step, such agents should be co-encapsulated into liposomes to combine the synergistic immune effects of both agents with the classical benefits associated when using liposomal formulations. There are several liposomal formulations that have shown therapeutic benefits by modulating the TME and tumour-associated immune response. Further research will allow the identification of liposomal formulations and combinations, thereof, with improved efficacy against cancer.

## Figures and Tables

**Table 1:** Key conditions of the tumour microenvironment.

Conditions of the TME	Effect
Hypoxia	Limited oxygen supply to cancer cells
Acidity	Drop in environmental pH
Neovascularisation	Formation of new, irregular blood vessels
Inflammation	Similar state to chronic inflammation
Dense extracellular matrix	Physical barriers preventing lymphocyte infiltration
Immune cells suppressing cancer	Activation of antitumour immune response (dendritic cells, CD8^+^ T-cells, NK cells)
Immune cells promoting cancer	Inhibition of antitumour immune response (T_reg_, tumour-associated macrophages, myeloid-derived suppressor cells)

**Table 2:** Approved liposomal formulations for cancer treatment and their indications.

Liposomal Formulation	Encapsulated Drug	Indication
DepoCyt™	Cytarabine	Lymphomatous meningitis, intrathecal treatment
Vyxeos™	Cytarabine + Daunorubicin	Newly-diagnosed therapy-related acute myeloid leukaemia in adults Acute myeloid leukaemia with myelodysplasia-related changes in adults
DaunoXome™	Daunorubicin	Advanced AIDS-related Kaposi’s sarcoma (discontinued by the U S Food and Drug Administration in 2016)
Caelyx™	Doxorubicin	Metastatic breast cancer where there is an increased cardiac risk associated with conventional doxorubicin Advanced ovarian carcinoma, in women who failed standard first-line platinum- and paclitaxel-based chemotherapy AIDS-related Kaposi’s sarcoma, in patients with low CD4 counts and extensive mucocutaneous or visceral disease, whose disease progressed despite therapy, or who are intolerant to prior systemic combination therapy comprising of at least two of the following agents: a vinca alkaloid, bleomycin, and doxorubicin (or another anthracycline)
Doxil™	Doxorubicin	Ovarian cancer after failure of platinum-based chemotherapy AIDS-related Kaposi’s sarcoma after failure of prior combination chemotherapy, or intolerance to such therapy Multiple myeloma that did not previously receive bortezomib and received at least one prior therapy, in combination with bortezomib
Myocet™	Doxorubicin	Metastatic breast cancer, first-line treatment in adult women, in combination with cyclophosphamide
Onivyde™	Irinotecan	Metastatic adenocarcinoma of the pancreas after disease progression following gemcitabine-based therapy, in combination with 5-fluorouracil and leucovorin
Mepact™	Mifamurtide	High-grade resectable non-metastatic osteosarcoma, after macroscopically complete surgical resection in children, adolescents, and young adults, in combination with post-operative multiagent chemotherapy
Marquibo™	Vincristine	Philadelphia chromosome-negative acute lymphoblastic leukaemia, in adult patients in second or greater relapse, or whose disease has progressed following two or more anti-leukaemia therapies

The information relative to the indications was obtained from the prescribing information of the respective liposomal formulations as of 2018.

**Table 3:** Liposomal formulations that modulate the tumour microenvironment (TME) and are used in the clinic.

Liposomal Formulation	Activity	Mechanism of Action
Liposomal doxorubicin	Inhibition of angiogenesis	Direct cytotoxicity on tumour-associated endothelial cells Downregulation of VEGF
	Increase of antitumour immune response	Reduction of T_reg_, Increase of CD8^+^ T-cells Upregulation of CD80, MHC-I, and Fas
Liposomal irinotecan	Inhibition of angiogenesis	Direct cytotoxicity on tumour-associated endothelial cells Downregulation of VEGF and IL8, upregulation of TIMP-1
Liposomal paclitaxel	Inhibition of angiogenesis	Direct cytotoxicity on tumour-associated endothelial cells
Liposomal mifamurtide	Increase of antitumour immune response	Stimulation of monocytes and macrophages Increased secretion of TNF-α, IL-1, IL-6, IL-8, nitric oxide, prostaglandin E2, and prostaglandin D2 Increased expression of LFA-1, ICAM-1, and HLA-DR

## References

[1] Siegel R.L., Miller K.D., Jemal A. (2018). Cancer statistics, 2018. CA Cancer J. Clin..

[2] Gotwals P., Cameron S., Cipolletta D., Cremasco V., Crystal A., Hewes B., Mueller B., Quaratino S., Sabatos-Peyton C., Petruzzelli L. (2017). Prospects for combining targeted and conventional cancer therapy with immunotherapy. Nat. Rev. Cancer.

[3] Miller K.D., Siegel R.L., Lin C.C., Mariotto A.B., Kramer J.L., Rowland J.H., Stein K.D., Alteri R., Jemal A. (2016). Cancer treatment and survivorship statistics, 2016. CA Cancer J. Clin..

[4] Ramos P., Bentires-Alj M. (2015). Mechanism-based cancer therapy: Resistance to therapy, therapy for resistance. Oncogene.

[5] Massague J., Batlle E., Gomis R.R. (2017). Understanding the molecular mechanisms driving metastasis. Mol. Oncol..

[6] Lambert A.W., Pattabiraman D.R., Weinberg R.A. (2017). Emerging biological principles of metastasis. Cell.

[7] Hui L., Chen Y. (2015). Tumor microenvironment: Sanctuary of the devil. Cancer Lett..

[8] Bozic I., Antal T., Ohtsuki H., Carter H., Kim D., Chen S., Karchin R., Kinzler K.W., Vogelstein B., Nowak M.A. (2010). Accumulation of driver and passenger mutations during tumor progression. Proc. Natl. Acad. Sci. USA.

[9] Hanahan D., Coussens L.M. (2012). Accessories to the crime: Functions of cells recruited to the tumor microenvironment. Cancer Cell.

[10] Balkwill F.R., Capasso M., Hagemann T. (2012). The tumor microenvironment at a glance. J. Cell Sci..

[11] Klemm F., Joyce J.A. (2015). Microenvironmental regulation of therapeutic response in cancer. Trends Cell Biol..

[12] Saggar J.K., Yu M., Tan Q., Tannock I.F. (2013). The tumor microenvironment and strategies to improve drug distribution. Front. Oncol..

[13] Chen F., Zhuang X., Lin L., Yu P., Wang Y., Shi Y., Hu G., Sun Y. (2015). New horizons in tumor microenvironment biology: Challenges and opportunities. BMC Med..

[14] Allen T.M., Cullis P.R. (2013). Liposomal drug delivery systems: From concept to clinical applications. Adv. Drug. Deliv. Rev..

[15] Gilabert-Oriol R., Chernov L., Deyell R.J., Bally M.B. (2018). Developing liposomal nanomedicines for treatment of patients with neuroblastoma. Lipid Nanocarriers for Drug Targeting.

[16] Danhier F., Feron O., Preat V. (2010). To exploit the tumor microenvironment: Passive and active tumor targeting of nanocarriers for anti-cancer drug delivery. J. Control Release.

[17] Maeda H. (2015). Toward a full understanding of the epr effect in primary and metastatic tumors as well as issues related to its heterogeneity. Adv. Drug Deliv. Rev..

[18] Zhao G., Rodriguez B.L. (2013). Molecular targeting of liposomal nanoparticles to tumor microenvironment. Int. J. Nanomed..

[19] Bertrand N., Wu J., Xu X., Kamaly N., Farokhzad O.C. (2014). Cancer nanotechnology: The impact of passive and active targeting in the era of modern cancer biology. Adv. Drug. Deliv. Rev..

[20] You J.S., Jones P.A. (2012). Cancer genetics and epigenetics: Two sides of the same coin?. Cancer Cell.

[21] Hanahan D., Weinberg R.A. (2011). Hallmarks of cancer: The next generation. Cell.

[22] Mbeunkui F., Johann D.J. (2009). Cancer and the tumor microenvironment: A review of an essential relationship. Cancer Chemother. Pharmacol..

[23] Quail D.F., Joyce J.A. (2013). Microenvironmental regulation of tumor progression and metastasis. Nat. Med..

[24] Jubb A.M., Buffa F.M., Harris A.L. (2010). Assessment of tumour hypoxia for prediction of response to therapy and cancer prognosis. J. Cell Mol. Med..

[25] Carmeliet P., Jain R.K. (2000). Angiogenesis in cancer and other diseases. Nature.

[26] Masson N., Ratcliffe P.J. (2014). Hypoxia signaling pathways in cancer metabolism: The importance of co-selecting interconnected physiological pathways. Cancer Metab..

[27] Wilson W.R., Hay M.P. (2011). Targeting hypoxia in cancer therapy. Nat. Rev. Cancer.

[28] Kato Y., Ozawa S., Miyamoto C., Maehata Y., Suzuki A., Maeda T., Baba Y. (2013). Acidic extracellular microenvironment and cancer. Cancer Cell Int..

[29] Webb B.A., Chimenti M., Jacobson M.P., Barber D.L. (2011). Dysregulated ph: A perfect storm for cancer progression. Nat. Rev. Cancer.

[30] Estrella V., Chen T., Lloyd M., Wojtkowiak J., Cornnell H.H., Ibrahim-Hashim A., Bailey K., Balagurunathan Y., Rothberg J.M., Sloane B.F. (2013). Acidity generated by the tumor microenvironment drives local invasion. Cancer Res..

[31] Huber V., Camisaschi C., Berzi A., Ferro S., Lugini L., Triulzi T., Tuccitto A., Tagliabue E., Castelli C., Rivoltini L. (2017). Cancer acidity: An ultimate frontier of tumor immune escape and a novel target of immunomodulation. Semin. Cancer Biol..

[32] Feng L., Cheng L., Dong Z., Tao D., Barnhart T.E., Cai W., Chen M., Liu Z. (2017). Theranostic liposomes with hypoxia-activated prodrug to effectively destruct hypoxic tumors post-photodynamic therapy. ACS Nano.

[33] Zhao Y., Ren W., Zhong T., Zhang S., Huang D., Guo Y., Yao X., Wang C., Zhang W.Q., Zhang X. (2016). Tumor-specific ph-responsive peptide-modified ph-sensitive liposomes containing doxorubicin for enhancing glioma targeting and anti-tumor activity. J. Control Release.

[34] Landskron G., De la Fuente M., Thuwajit P., Thuwajit C., Hermoso M.A. (2014). Chronic inflammation and cytokines in the tumor microenvironment. J. Immunol. Res..

[35] Weis S.M., Cheresh D.A. (2011). Tumor angiogenesis: Molecular pathways and therapeutic targets. Nat. Med..

[36] Bussard K.M., Mutkus L., Stumpf K., Gomez-Manzano C., Marini F.C. (2016). Tumor-associated stromal cells as key contributors to the tumor microenvironment. Breast Cancer Res..

[37] Ronca R., Benkheil M., Mitola S., Struyf S., Liekens S. (2017). Tumor angiogenesis revisited: Regulators and clinical implications. Med. Res. Rev..

[38] Johnson L.A., Clasper S., Holt A.P., Lalor P.F., Baban D., Jackson D.G. (2006). An inflammation-induced mechanism for leukocyte transmigration across lymphatic vessel endothelium. J. Exp. Med..

[39] Harlin H., Meng Y., Peterson A.C., Zha Y., Tretiakova M., Slingluff C., McKee M., Gajewski T.F. (2009). Chemokine expression in melanoma metastases associated with cd8+ t-cell recruitment. Cancer Res..

[40] Kerbel R.S., Kamen B.A. (2004). The anti-angiogenic basis of metronomic chemotherapy. Nat. Rev. Cancer.

[41] Verreault M., Strutt D., Masin D., Anantha M., Yung A., Kozlowski P., Waterhouse D., Bally M.B., Yapp D.T. (2011). Vascular normalization in orthotopic glioblastoma following intravenous treatment with lipid-based nanoparticulate formulations of irinotecan (irinophore c), doxorubicin (caelyx(r)) or vincristine. BMC Cancer.

[42] Jayson G.C., Kerbel R., Ellis L.M., Harris A.L. (2016). Antiangiogenic therapy in oncology: Current status and future directions. Lancet.

[43] Schumacher T.N., Schreiber R.D. (2015). Neoantigens in cancer immunotherapy. Science.

[44] Teng M.W., Ngiow S.F., Ribas A., Smyth M.J. (2015). Classifying cancers based on t-cell infiltration and pd-l1. Cancer Res..

[45] Xing F., Saidou J., Watabe K. (2010). Cancer associated fibroblasts (cafs) in tumor microenvironment. Front. Biosci. (Landmark Ed.).

[46] Bremnes R.M., Donnem T., Al-Saad S., Al-Shibli K., Andersen S., Sirera R., Camps C., Marinez I., Busund L.T. (2011). The role of tumor stroma in cancer progression and prognosis: Emphasis on carcinoma-associated fibroblasts and non-small cell lung cancer. J. Thorac. Oncol..

[47] Heldin C.H., Rubin K., Pietras K., Ostman A. (2004). High interstitial fluid pressure—An obstacle in cancer therapy. Nat. Rev. Cancer.

[48] Dirat B., Bochet L., Dabek M., Daviaud D., Dauvillier S., Majed B., Wang Y.Y., Meulle A., Salles B., Le Gonidec S. (2011). Cancer-associated adipocytes exhibit an activated phenotype and contribute to breast cancer invasion. Cancer Res..

[49] Nieman K.M., Kenny H.A., Penicka C.V., Ladanyi A., Buell-Gutbrod R., Zillhardt M.R., Romero I.L., Carey M.S., Mills G.B., Hotamisligil G.S. (2011). Adipocytes promote ovarian cancer metastasis and provide energy for rapid tumor growth. Nat. Med..

[50] Lu P., Takai K., Weaver V.M., Werb Z. (2011). Extracellular matrix degradation and remodeling in development and disease. Cold Spring Harb. Perspect. Biol..

[51] Gajewski T.F., Schreiber H., Fu Y.X. (2013). Innate and adaptive immune cells in the tumor microenvironment. Nat. Immunol..

[52] Melero I., Gaudernack G., Gerritsen W., Huber C., Parmiani G., Scholl S., Thatcher N., Wagstaff J., Zielinski C., Faulkner I. (2014). Therapeutic vaccines for cancer: An overview of clinical trials. Nat. Rev. Clin. Oncol..

[53] Ma Y., Adjemian S., Mattarollo S.R., Yamazaki T., Aymeric L., Yang H., Portela Catani J.P., Hannani D., Duret H., Steegh K. (2013). Anticancer chemotherapy-induced intratumoral recruitment and differentiation of antigen-presenting cells. Immunity.

[54] Galluzzi L., Buque A., Kepp O., Zitvogel L., Kroemer G. (2017). Immunogenic cell death in cancer and infectious disease. Nat. Rev. Immunol..

[55] Golden E.B., Frances D., Pellicciotta I., Demaria S., Helen Barcellos-Hoff M., Formenti S.C. (2014). Radiation fosters dose-dependent and chemotherapy-induced immunogenic cell death. Oncoimmunology.

[56] Janicka M., Gubernator J. (2017). Use of nanotechnology for improved pharmacokinetics and activity of immunogenic cell death inducers used in cancer chemotherapy. Expert. Opin. Drug Deliv..

[57] Galon J., Costes A., Sanchez-Cabo F., Kirilovsky A., Mlecnik B., Lagorce-Pages C., Tosolini M., Camus M., Berger A., Wind P. (2006). Type, density, and location of immune cells within human colorectal tumors predict clinical outcome. Science.

[58] Mahmoud S.M., Paish E.C., Powe D.G., Macmillan R.D., Grainge M.J., Lee A.H., Ellis I.O., Green A.R. (2011). Tumor-infiltrating cd8+ lymphocytes predict clinical outcome in breast cancer. J. Clin. Oncol..

[59] Azimi F., Scolyer R.A., Rumcheva P., Moncrieff M., Murali R., McCarthy S.W., Saw R.P., Thompson J.F. (2012). Tumor-infiltrating lymphocyte grade is an independent predictor of sentinel lymph node status and survival in patients with cutaneous melanoma. J. Clin. Oncol..

[60] Rusakiewicz S., Semeraro M., Sarabi M., Desbois M., Locher C., Mendez R., Vimond N., Concha A., Garrido F., Isambert N. (2013). Immune infiltrates are prognostic factors in localized gastrointestinal stromal tumors. Cancer Res..

[61] Sancho D., Joffre O.P., Keller A.M., Rogers N.C., Martinez D., Hernanz-Falcon P., Rosewell I., e Sousa C.R. (2009). Identification of a dendritic cell receptor that couples sensing of necrosis to immunity. Nature.

[62] Poulin L.F., Salio M., Griessinger E., Anjos-Afonso F., Craciun L., Chen J.L., Keller A.M., Joffre O., Zelenay S., Nye E. (2010). Characterization of human dngr-1+ bdca3+ leukocytes as putative equivalents of mouse cd8alpha+ dendritic cells. J. Exp. Med..

[63] Morvan M.G., Lanier L.L. (2016). Nk cells and cancer: You can teach innate cells new tricks. Nat. Rev. Cancer.

[64] Blank C., Mackensen A. (2007). Contribution of the pd-l1/pd-1 pathway to t-cell exhaustion: An update on implications for chronic infections and tumor evasion. Cancer Immunol. Immunother..

[65] Munn D.H., Mellor A.L. (2013). Indoleamine 2,3 dioxygenase and metabolic control of immune responses. Trends Immunol..

[66] Whiteside T.L. (2012). What are regulatory t cells (treg) regulating in cancer and why?. Semin. Cancer Biol..

[67] Halvorsen E.C., Mahmoud S.M., Bennewith K.L. (2014). Emerging roles of regulatory t cells in tumour progression and metastasis. Cancer Metast. Rev..

[68] Halvorsen E.C., Hamilton M.J., Young A., Wadsworth B.J., LePard N.E., Lee H.N., Firmino N., Collier J.L., Bennewith K.L. (2016). Maraviroc decreases ccl8-mediated migration of ccr5(+) regulatory t cells and reduces metastatic tumor growth in the lungs. Oncoimmunology.

[69] Crespo J., Sun H., Welling T.H., Tian Z., Zou W. (2013). T cell anergy, exhaustion, senescence, and stemness in the tumor microenvironment. Curr. Opin. Immunol..

[70] Hamilton M.J., Bosiljcic M., Lepard N.E., Halvorsen E.C., Ho V.W., Banath J.P., Krystal G., Bennewith K.L. (2014). Macrophages are more potent immune suppressors ex vivo than immature myeloid-derived suppressor cells induced by metastatic murine mammary carcinomas. J. Immunol..

[71] Quatromoni J.G., Eruslanov E. (2012). Tumor-associated macrophages: Function, phenotype, and link to prognosis in human lung cancer. Am. J. Transl. Res..

[72] Noy R., Pollard J.W. (2014). Tumor-associated macrophages: From mechanisms to therapy. Immunity.

[73] Gabrilovich D.I. (2017). Myeloid-derived suppressor cells. Cancer Immunol. Res..

[74] Markovic S.N., Kumar A.B., Dong H., Markovic S.N. (2018). Therapeutic targets of fda-approved immunotherapies in oncology. The Basics of Cancer Immunotherapy.

[75] Jiang T., Zhou C., Ren S. (2016). Role of il-2 in cancer immunotherapy. Oncoimmunology.

[76] Zheng P.P., Kros J.M., Li J. (2018). Approved car t cell therapies: Ice bucket challenges on glaring safety risks and long-term impacts. Drug Discov. Today.

[77] Kosti P., Maher J., Arnold J.N. (2018). Perspectives on chimeric antigen receptor t-cell immunotherapy for solid tumors. Front. Immunol..

[78] Marin-Acevedo J.A., Soyano A.E., Dholaria B., Knutson K.L., Lou Y. (2018). Cancer immunotherapy beyond immune checkpoint inhibitors. J. Hematol. Oncol..

[79] Showalter A., Limaye A., Oyer J.L., Igarashi R., Kittipatarin C., Copik A.J., Khaled A.R. (2017). Cytokines in immunogenic cell death: Applications for cancer immunotherapy. Cytokine.

[80] Gubin M.M., Schreiber R.D. (2015). The odds of immunotherapy success. Science.

[81] Tesniere A., Schlemmer F., Boige V., Kepp O., Martins I., Ghiringhelli F., Aymeric L., Michaud M., Apetoh L., Barault L. (2010). Immunogenic death of colon cancer cells treated with oxaliplatin. Oncogene.

[82] Obeid M., Tesniere A., Ghiringhelli F., Fimia G.M., Apetoh L., Perfettini J.L., Castedo M., Mignot G., Panaretakis T., Casares N. (2007). Calreticulin exposure dictates the immunogenicity of cancer cell death. Nat. Med..

[83] Martins I., Wang Y., Michaud M., Ma Y., Sukkurwala A.Q., Shen S., Kepp O., Metivier D., Galluzzi L., Perfettini J.L. (2014). Molecular mechanisms of atp secretion during immunogenic cell death. Cell Death Differ..

[84] Bezu L., Gomes-de-Silva L.C., Dewitte H., Breckpot K., Fucikova J., Spisek R., Galluzzi L., Kepp O., Kroemer G. (2015). Combinatorial strategies for the induction of immunogenic cell death. Front. Immunol..

[85] Garg A.D., Galluzzi L., Apetoh L., Baert T., Birge R.B., Bravo-San Pedro J.M., Breckpot K., Brough D., Chaurio R., Cirone M. (2015). Molecular and translational classifications of damps in immunogenic cell death. Front. Immunol..

[86] Krysko D.V., Garg A.D., Kaczmarek A., Krysko O., Agostinis P., Vandenabeele P. (2012). Immunogenic cell death and damps in cancer therapy. Nat. Rev. Cancer.

[87] Vacchelli E., Galluzzi L., Fridman W.H., Galon J., Sautes-Fridman C., Tartour E., Kroemer G. (2012). Trial watch: Chemotherapy with immunogenic cell death inducers. Oncoimmunology.

[88] Kepp O., Menger L., Vacchelli E., Locher C., Adjemian S., Yamazaki T., Martins I., Sukkurwala A.Q., Michaud M., Senovilla L. (2013). Crosstalk between er stress and immunogenic cell death. Cytokine Growth Factor Rev..

[89] Panaretakis T., Kepp O., Brockmeier U., Tesniere A., Bjorklund A.C., Chapman D.C., Durchschlag M., Joza N., Pierron G., van Endert P. (2009). Mechanisms of pre-apoptotic calreticulin exposure in immunogenic cell death. EMBO J..

[90] Gardai S.J., McPhillips K.A., Frasch S.C., Janssen W.J., Starefeldt A., Murphy-Ullrich J.E., Bratton D.L., Oldenborg P.A., Michalak M., Henson P.M. (2005). Cell-surface calreticulin initiates clearance of viable or apoptotic cells through trans-activation of lrp on the phagocyte. Cell.

[91] Gebremeskel S., Johnston B. (2015). Concepts and mechanisms underlying chemotherapy induced immunogenic cell death: Impact on clinical studies and considerations for combined therapies. Oncotarget.

[92] Di Virgilio F. (2016). P2rx7: A receptor with a split personality in inflammation and cancer. Mol. Cell Oncol..

[93] Di Giovine F.S., Poole S., Situnayake R.D., Wadhwa M., Duff G.W. (1990). Absence of correlations between indices of systemic inflammation and synovial fluid interleukin 1 (alpha and beta) in rheumatic diseases. Rheumatol. Int..

[94] Tang D., Kang R., Livesey K.M., Cheh C.W., Farkas A., Loughran P., Hoppe G., Bianchi M.E., Tracey K.J., Zeh H.J. (2010). Endogenous hmgb1 regulates autophagy. J. Cell Biol..

[95] Yang H., Wang H., Czura C.J., Tracey K.J. (2005). The cytokine activity of hmgb1. J. Leukoc. Biol..

[96] Kepp O., Tesniere A., Schlemmer F., Michaud M., Senovilla L., Zitvogel L., Kroemer G. (2009). Immunogenic cell death modalities and their impact on cancer treatment. Apoptosis.

[97] Thorburn J., Horita H., Redzic J., Hansen K., Frankel A.E., Thorburn A. (2009). Autophagy regulates selective hmgb1 release in tumor cells that are destined to die. Cell Death Differ..

[98] Kepp O., Senovilla L., Vitale I., Vacchelli E., Adjemian S., Agostinis P., Apetoh L., Aranda F., Barnaba V., Bloy N. (2014). Consensus guidelines for the detection of immunogenic cell death. Oncoimmunology.

[99] Yamazaki T., Hannani D., Poirier-Colame V., Ladoire S., Locher C., Sistigu A., Prada N., Adjemian S., Catani J.P., Freudenberg M. (2014). Defective immunogenic cell death of hmgb1-deficient tumors: Compensatory therapy with tlr4 agonists. Cell Death Differ..

[100] Tesniere A., Panaretakis T., Kepp O., Apetoh L., Ghiringhelli F., Zitvogel L., Kroemer G. (2008). Molecular characteristics of immunogenic cancer cell death. Cell Death Differ..

[101] Golden E.B., Pellicciotta I., Demaria S., Barcellos-Hoff M.H., Formenti S.C. (2012). The convergence of radiation and immunogenic cell death signaling pathways. Front. Oncol..

[102] Burton D.G., Faragher R.G. (2015). Cellular senescence: From growth arrest to immunogenic conversion. Age (Dordr).

[103] Harrington K.J., Billingham L.J., Brunner T.B., Burnet N.G., Chan C.S., Hoskin P., Mackay R.I., Maughan T.S., Macdougall J., McKenna W.G. (2011). Guidelines for preclinical and early phase clinical assessment of novel radiosensitisers. Br. J. Cancer.

[104] Dolmans D.E., Fukumura D., Jain R.K. (2003). Photodynamic therapy for cancer. Nat. Rev. Cancer.

[105] Stone H.B., Peters L.J., Milas L. (1979). Effect of host immune capability on radiocurability and subsequent transplantability of a murine fibrosarcoma. J. Natl. Cancer Inst..

[106] Lee Y., Auh S.L., Wang Y., Burnette B., Wang Y., Meng Y., Beckett M., Sharma R., Chin R., Tu T. (2009). Therapeutic effects of ablative radiation on local tumor require cd8+ t cells: Changing strategies for cancer treatment. Blood.

[107] Pitt J.M., Kroemer G., Zitvogel L. (2017). Immunogenic and non-immunogenic cell death in the tumor microenvironment. Adv. Exp. Med. Biol..

[108] Jurin M., Suit H.D. (1972). In vivo and in vitro studies of the influence of the immune status of c3hf-bu mice on the effectiveness of local irradiation of a methylcholanthrene-induced fibrosarcoma. Cancer Res..

[109] Obeid M., Panaretakis T., Joza N., Tufi R., Tesniere A., van Endert P., Zitvogel L., Kroemer G. (2007). Calreticulin exposure is required for the immunogenicity of gamma-irradiation and uvc light-induced apoptosis. Cell Death Differ..

[110] Vanpouille-Box C., Alard A., Aryankalayil M.J., Sarfraz Y., Diamond J.M., Schneider R.J., Inghirami G., Coleman C.N., Formenti S.C., Demaria S. (2017). DNA exonuclease trex1 regulates radiotherapy-induced tumour immunogenicity. Nat. Commun..

[111] Ko A., Kanehisa A., Martins I., Senovilla L., Chargari C., Dugue D., Marino G., Kepp O., Michaud M., Perfettini J.L. (2014). Autophagy inhibition radiosensitizes in vitro, yet reduces radioresponses in vivo due to deficient immunogenic signalling. Cell Death Differ..

[112] Apetoh L., Ghiringhelli F., Tesniere A., Obeid M., Ortiz C., Criollo A., Mignot G., Maiuri M.C., Ullrich E., Saulnier P. (2007). Toll-like receptor 4-dependent contribution of the immune system to anticancer chemotherapy and radiotherapy. Nat. Med..

[113] Shaverdian N., Lisberg A.E., Bornazyan K., Veruttipong D., Goldman J.W., Formenti S.C., Garon E.B., Lee P. (2017). Previous radiotherapy and the clinical activity and toxicity of pembrolizumab in the treatment of non-small-cell lung cancer: A secondary analysis of the keynote-001 phase 1 trial. Lancet Oncol..

[114] Deng L., Liang H., Burnette B., Beckett M., Darga T., Weichselbaum R.R., Fu Y.X. (2014). Irradiation and anti-pd-l1 treatment synergistically promote antitumor immunity in mice. J. Clin. Investig..

[115] Dewan M.Z., Galloway A.E., Kawashima N., Dewyngaert J.K., Babb J.S., Formenti S.C., Demaria S. (2009). Fractionated but not single-dose radiotherapy induces an immune-mediated abscopal effect when combined with anti-ctla-4 antibody. Clin. Cancer Res..

[116] Rothkamm K., Kruger I., Thompson L.H., Lobrich M. (2003). Pathways of DNA double-strand break repair during the mammalian cell cycle. Mol. Cell Biol..

[117] Bao S., Wu Q., McLendon R.E., Hao Y., Shi Q., Hjelmeland A.B., Dewhirst M.W., Bigner D.D., Rich J.N. (2006). Glioma stem cells promote radioresistance by preferential activation of the DNA damage response. Nature.

[118] Hill R.P. (2017). The changing paradigm of tumour response to irradiation. Br. J. Radiol..

[119] Shenoy M.A., Asquith J.C., Adams G.E., Micheal B.D., Watts M.E. (1975). Time-resolved oxygen effects in irradiated bacteria and mammalian cells: A rapid-mix study. Radiat. Res..

[120] Olive P.L., Banath J.P. (2004). Phosphorylation of histone h2ax as a measure of radiosensitivity. Int. J. Radiat. Oncol. Biol. Phys..

[121] Brizel D.M., Dodge R.K., Clough R.W., Dewhirst M.W. (1999). Oxygenation of head and neck cancer: Changes during radiotherapy and impact on treatment outcome. Radiother. Oncol..

[122] Hockel M., Schlenger K., Mitze M., Schaffer U., Vaupel P. (1996). Hypoxia and radiation response in human tumors. Semin. Radiat. Oncol..

[123] Barcellos-Hoff M.H., Derynck R., Tsang M.L., Weatherbee J.A. (1994). Transforming growth factor-beta activation in irradiated murine mammary gland. J. Clin. Investig..

[124] Liang H., Deng L., Hou Y., Meng X., Huang X., Rao E., Zheng W., Mauceri H., Mack M., Xu M. (2017). Host sting-dependent mdsc mobilization drives extrinsic radiation resistance. Nat. Commun..

[125] Vanpouille-Box C., Diamond J.M., Pilones K.A., Zavadil J., Babb J.S., Formenti S.C., Barcellos-Hoff M.H., Demaria S. (2015). Tgfbeta is a master regulator of radiation therapy-induced antitumor immunity. Cancer Res..

[126] Dudek A.M., Garg A.D., Krysko D.V., De Ruysscher D., Agostinis P. (2013). Inducers of immunogenic cancer cell death. Cytokine Growth Factor Rev..

[127] Spisek R., Charalambous A., Mazumder A., Vesole D.H., Jagannath S., Dhodapkar M.V. (2007). Bortezomib enhances dendritic cell (dc)-mediated induction of immunity to human myeloma via exposure of cell surface heat shock protein 90 on dying tumor cells: Therapeutic implications. Blood.

[128] Chen H.M., Wang P.H., Chen S.S., Wen C.C., Chen Y.H., Yang W.C., Yang N.S. (2012). Shikonin induces immunogenic cell death in tumor cells and enhances dendritic cell-based cancer vaccine. Cancer Immunol. Immunother..

[129] Garg A.D., Krysko D.V., Verfaillie T., Kaczmarek A., Ferreira G.B., Marysael T., Rubio N., Firczuk M., Mathieu C., Roebroek A.J. (2012). A novel pathway combining calreticulin exposure and atp secretion in immunogenic cancer cell death. EMBO J..

[130] Miyamoto S., Inoue H., Nakamura T., Yamada M., Sakamoto C., Urata Y., Okazaki T., Marumoto T., Takahashi A., Takayama K. (2012). Coxsackievirus b3 is an oncolytic virus with immunostimulatory properties that is active against lung adenocarcinoma. Cancer Res..

[131] Martins I., Tesniere A., Kepp O., Michaud M., Schlemmer F., Senovilla L., Seror C., Metivier D., Perfettini J.L., Zitvogel L. (2009). Chemotherapy induces atp release from tumor cells. Cell Cycle.

[132] Mihailidou C., Chatzistamou I., Papavassiliou A.G., Kiaris H. (2015). Improvement of chemotherapeutic drug efficacy by endoplasmic reticulum stress. Endocr. Relat. Cancer.

[133] Martins I., Kepp O., Schlemmer F., Adjemian S., Tailler M., Shen S., Michaud M., Menger L., Gdoura A., Tajeddine N. (2011). Restoration of the immunogenicity of cisplatin-induced cancer cell death by endoplasmic reticulum stress. Oncogene.

[134] Aranda F., Bloy N., Pesquet J., Petit B., Chaba K., Sauvat A., Kepp O., Khadra N., Enot D., Pfirschke C. (2015). Immune-dependent antineoplastic effects of cisplatin plus pyridoxine in non-small-cell lung cancer. Oncogene.

[135] Aranda F., Bloy N., Galluzzi L., Kroemer G., Senovilla L. (2014). Vitamin b6 improves the immunogenicity of cisplatin-induced cell death. Oncoimmunology.

[136] Zhao T., Ren H., Jia L., Chen J., Xin W., Yan F., Li J., Wang X., Gao S., Qian D. (2015). Inhibition of hif-1alpha by px-478 enhances the anti-tumor effect of gemcitabine by inducing immunogenic cell death in pancreatic ductal adenocarcinoma. Oncotarget.

[137] Michaud M., Martins I., Sukkurwala A.Q., Adjemian S., Ma Y., Pellegatti P., Shen S., Kepp O., Scoazec M., Mignot G. (2011). Autophagy-dependent anticancer immune responses induced by chemotherapeutic agents in mice. Science.

[138] Gardner E.R., Dahut W.L., Scripture C.D., Jones J., Aragon-Ching J.B., Desai N., Hawkins M.J., Sparreboom A., Figg W.D. (2008). Randomized crossover pharmacokinetic study of solvent-based paclitaxel and nab-paclitaxel. Clin. Cancer Res..

[139] Cortes J., Saura C. (2010). Nanoparticle albumin-bound (nab™)-paclitaxel: Improving efficacy and tolerability by targeted drug delivery in metastatic breast cancer. EJC Suppl..

[140] Siegler E.L., Kim Y.J., Wang P. (2016). Nanomedicine targeting the tumor microenvironment: Therapeutic strategies to inhibit angiogenesis, remodel matrix, and modulate immune responses. J. Cell Immunother..

[141] Anselmo A.C., Mitragotri S. (2016). Nanoparticles in the clinic. Bioeng. Transl. Med..

[142] Madden T.D., Harrigan P.R., Tai L.C., Bally M.B., Mayer L.D., Redelmeier T.E., Loughrey H.C., Tilcock C.P., Reinish L.W., Cullis P.R. (1990). The accumulation of drugs within large unilamellar vesicles exhibiting a proton gradient: A survey. Chem. Phys. Lipids.

[143] Wehbe M., Anantha M., Backstrom I., Leung A., Chen K., Malhotra A., Edwards K., Bally M.B. (2016). Nanoscale reaction vessels designed for synthesis of copper-drug complexes suitable for preclinical development. PLoS ONE.

[144] Klibanov A.L., Maruyama K., Torchilin V.P., Huang L. (1990). Amphipathic polyethyleneglycols effectively prolong the circulation time of liposomes. FEBS Lett..

[145] Allen C., Dos Santos N., Gallagher R., Chiu G.N., Shu Y., Li W.M., Johnstone S.A., Janoff A.S., Mayer L.D., Webb M.S. (2002). Controlling the physical behavior and biological performance of liposome formulations through use of surface grafted poly(ethylene glycol). Biosci. Rep..

[146] Senior J., Delgado C., Fisher D., Tilcock C., Gregoriadis G. (1991). Influence of surface hydrophilicity of liposomes on their interaction with plasma protein and clearance from the circulation: Studies with poly(ethylene glycol)-coated vesicles. Biochim. Biophys. Acta.

[147] Allen T.M. (1994). Long-circulating (sterically stabilized) liposomes for targeted drug delivery. Trends Pharmacol. Sci..

[148] Ishida T., Kiwada H. (2008). Accelerated blood clearance (abc) phenomenon upon repeated injection of pegylated liposomes. Int. J. Pharm..

[149] Koide H., Asai T., Hatanaka K., Akai S., Ishii T., Kenjo E., Ishida T., Kiwada H., Tsukada H., Oku N. (2010). T cell-independent b cell response is responsible for abc phenomenon induced by repeated injection of pegylated liposomes. Int. J. Pharm..

[150] Wang X., Ishida T., Kiwada H. (2007). Anti-peg igm elicited by injection of liposomes is involved in the enhanced blood clearance of a subsequent dose of pegylated liposomes. J. Control Release.

[151] Xu X., Wang L., Xu H.Q., Huang X.E., Qian Y.D., Xiang J. (2013). Clinical comparison between paclitaxel liposome (lipusu(r)) and paclitaxel for treatment of patients with metastatic gastric cancer. Asian Pac. J. Cancer Prev..

[152] Safra T., Muggia F., Jeffers S., Tsao-Wei D.D., Groshen S., Lyass O., Henderson R., Berry G., Gabizon A. (2000). Pegylated liposomal doxorubicin (doxil): Reduced clinical cardiotoxicity in patients reaching or exceeding cumulative doses of 500 mg/m^2^. Ann. Oncol..

[153] Swenson C.E., Perkins W.R., Roberts P., Janoff A.S. (2001). Liposome technology and the development of myocet™ (liposomal doxorubicin citrate). Breast.

[154] Boman N.L., Bally M.B., Cullis P.R., Mayer L.D., Webb M.S. (1995). Encapsulation of vincristine in liposomes reduces its toxicity and improves its anti-tumor efficacy. J. Liposome Res..

[155] Matsumura Y., Maeda H. (1986). A new concept for macromolecular therapeutics in cancer chemotherapy: Mechanism of tumoritropic accumulation of proteins and the antitumor agent smancs. Cancer Res..

[156] Prabhakar U., Maeda H., Jain R.K., Sevick-Muraca E.M., Zamboni W., Farokhzad O.C., Barry S.T., Gabizon A., Grodzinski P., Blakey D.C. (2013). Challenges and key considerations of the enhanced permeability and retention effect for nanomedicine drug delivery in oncology. Cancer Res..

[157] Nakamura H., Fang J., Maeda H. (2015). Development of next-generation macromolecular drugs based on the epr effect: Challenges and pitfalls. Expert Opin. Drug Deliv..

[158] Nichols J.W., Bae Y.H. (2014). Epr: Evidence and fallacy. J. Control Release.

[159] Bae Y.H., Park K. (2011). Targeted drug delivery to tumors: Myths, reality and possibility. J. Control Release.

[160] Leu A.J., Berk D.A., Lymboussaki A., Alitalo K., Jain R.K. (2000). Absence of functional lymphatics within a murine sarcoma: A molecular and functional evaluation. Cancer Res..

[161] Browder T., Butterfield C.E., Kraling B.M., Shi B., Marshall B., O’Reilly M.S., Folkman J. (2000). Antiangiogenic scheduling of chemotherapy improves efficacy against experimental drug-resistant cancer. Cancer Res..

[162] Zhou R., Mazurchuk R., Straubinger R.M. (2002). Antivasculature effects of doxorubicin-containing liposomes in an intracranial rat brain tumor model. Cancer Res..

[163] Arnold R.D., Mager D.E., Slack J.E., Straubinger R.M. (2005). Effect of repetitive administration of doxorubicin-containing liposomes on plasma pharmacokinetics and drug biodistribution in a rat brain tumor model. Clin. Cancer Res..

[164] Ogawara K., Un K., Tanaka K., Higaki K., Kimura T. (2009). In vivo anti-tumor effect of peg liposomal doxorubicin (dox) in dox-resistant tumor-bearing mice: Involvement of cytotoxic effect on vascular endothelial cells. J. Control Release.

[165] Baker J.H., Lam J., Kyle A.H., Sy J., Oliver T., Co S.J., Dragowska W.H., Ramsay E., Anantha M., Ruth T.J. (2008). Irinophore c, a novel nanoformulation of irinotecan, alters tumor vascular function and enhances the distribution of 5-fluorouracil and doxorubicin. Clin. Cancer Res..

[166] Neijzen R., Wong M.Q., Gill N., Wang H., Karim T., Anantha M., Strutt D., Waterhouse D., Bally M.B., Tai I.T. (2015). Irinophore c, a lipid nanoparticulate formulation of irinotecan, improves vascular function, increases the delivery of sequentially administered 5-fu in ht-29 tumors, and controls tumor growth in patient derived xenografts of colon cancer. J. Control Release.

[167] Kunstfeld R., Wickenhauser G., Michaelis U., Teifel M., Umek W., Naujoks K., Wolff K., Petzelbauer P. (2003). Paclitaxel encapsulated in cationic liposomes diminishes tumor angiogenesis and melanoma growth in a “Humanized” Scid mouse model. J. Investig. Dermatol..

[168] Strieth S., Eichhorn M.E., Sauer B., Schulze B., Teifel M., Michaelis U., Dellian M. (2004). Neovascular targeting chemotherapy: Encapsulation of paclitaxel in cationic liposomes impairs functional tumor microvasculature. Int. J. Cancer.

[169] Strieth S., Nussbaum C.F., Eichhorn M.E., Fuhrmann M., Teifel M., Michaelis U., Berghaus A., Dellian M. (2008). Tumor-selective vessel occlusions by platelets after vascular targeting chemotherapy using paclitaxel encapsulated in cationic liposomes. Int. J. Cancer.

[170] Strieth S., Eichhorn M.E., Werner A., Sauer B., Teifel M., Michaelis U., Berghaus A., Dellian M. (2008). Paclitaxel encapsulated in cationic liposomes increases tumor microvessel leakiness and improves therapeutic efficacy in combination with cisplatin. Clin. Cancer Res..

[171] Ishida T., Harashima H., Kiwada H. (2002). Liposome clearance. Biosci. Rep..

[172] Rios-Doria J., Durham N., Wetzel L., Rothstein R., Chesebrough J., Holoweckyj N., Zhao W., Leow C.C., Hollingsworth R. (2015). Doxil synergizes with cancer immunotherapies to enhance antitumor responses in syngeneic mouse models. Neoplasia.

[173] Mayer L.D., Dougherty G., Harasym T.O., Bally M.B. (1997). The role of tumor-associated macrophages in the delivery of liposomal doxorubicin to solid murine fibrosarcoma tumors. J. Pharmacol. Exp. Ther..

[174] Banciu M., Schiffelers R.M., Storm G. (2008). Investigation into the role of tumor-associated macrophages in the antitumor activity of doxil. Pharm. Res..

[175] Alagkiozidis I., Facciabene A., Carpenito C., Benencia F., Jonak Z., Adams S., Carroll R.G., Gimotty P.A., Hammond R., Danet-Desnoyers G.A. (2009). Increased immunogenicity of surviving tumor cells enables cooperation between liposomal doxorubicin and il-18. J. Transl Med..

[176] Kheirolomoom A., Ingham E.S., Mahakian L.M., Tam S.M., Silvestrini M.T., Tumbale S.K., Foiret J., Hubbard N.E., Borowsky A.D., Murphy W.J. (2015). Cpg expedites regression of local and systemic tumors when combined with activatable nanodelivery. J. Control Release.

[177] Meyers P.A. (2009). Muramyl tripeptide (mifamurtide) for the treatment of osteosarcoma. Expert Rev. Anticancer Ther..

[178] Ando K., Mori K., Corradini N., Redini F., Heymann D. (2011). Mifamurtide for the treatment of nonmetastatic osteosarcoma. Expert Opin. Pharmacother..

[179] Pahl J.H., Kwappenberg K.M., Varypataki E.M., Santos S.J., Kuijjer M.L., Mohamed S., Wijnen J.T., van Tol M.J., Cleton-Jansen A.M., Egeler R.M. (2014). Macrophages inhibit human osteosarcoma cell growth after activation with the bacterial cell wall derivative liposomal muramyl tripeptide in combination with interferon-gamma. J. Exp. Clin. Cancer Res..

[180] Heymann M.-F., Heymann D. (2017). Immune environment and osteosarcoma. Osteosarcoma-Biology, Behavior and Mechanisms.

[181] Chamoto K., Takeshima T., Wakita D., Ohkuri T., Ashino S., Omatsu T., Shirato H., Kitamura H., Togashi Y., Nishimura T. (2009). Combination immunotherapy with radiation and cpg-based tumor vaccination for the eradication of radio- and immuno-resistant lung carcinoma cells. Cancer Sci..

[182] Suzuki Y., Wakita D., Chamoto K., Narita Y., Tsuji T., Takeshima T., Gyobu H., Kawarada Y., Kondo S., Akira S. (2004). Liposome-encapsulated cpg oligodeoxynucleotides as a potent adjuvant for inducing type 1 innate immunity. Cancer Res..

[183] Wakita D., Chamoto K., Zhang Y., Narita Y., Noguchi D., Ohnishi H., Iguchi T., Sakai T., Ikeda H., Nishimura T. (2006). An indispensable role of type-1 ifns for inducing ctl-mediated complete eradication of established tumor tissue by cpg-liposome co-encapsulated with model tumor antigen. Int. Immunol..

[184] Bennewith K.L., Dedhar S. (2011). Targeting hypoxic tumour cells to overcome metastasis. BMC Cancer.

[185] Liu H., Xie Y., Zhang Y., Cai Y., Li B., Mao H., Liu Y., Lu J., Zhang L., Yu R. (2017). Development of a hypoxia-triggered and hypoxic radiosensitized liposome as a doxorubicin carrier to promote synergetic chemo-/radio-therapy for glioma. Biomaterials.

[186] Zhang R., Song X., Liang C., Yi X., Song G., Chao Y., Yang Y., Yang K., Feng L., Liu Z. (2017). Catalase-loaded cisplatin-prodrug-constructed liposomes to overcome tumor hypoxia for enhanced chemo-radiotherapy of cancer. Biomaterials.

[187] Xu L., Qiu X., Zhang Y., Cao K., Zhao X., Wu J., Hu Y., Guo H. (2016). Liposome encapsulated perfluorohexane enhances radiotherapy in mice without additional oxygen supply. J. Transl. Med..

[188] Davies Cde L., Lundstrom L.M., Frengen J., Eikenes L., Bruland S.O., Kaalhus O., Hjelstuen M.H., Brekken C. (2004). Radiation improves the distribution and uptake of liposomal doxorubicin (caelyx) in human osteosarcoma xenografts. Cancer Res..

[189] Miller M.A., Chandra R., Cuccarese M.F., Pfirschke C., Engblom C., Stapleton S., Adhikary U., Kohler R.H., Mohan J.F., Pittet M.J. (2017). Radiation therapy primes tumors for nanotherapeutic delivery via macrophage-mediated vascular bursts. Sci. Transl. Med..

[190] Zolnik B.S., Gonzalez-Fernandez A., Sadrieh N., Dobrovolskaia M.A. (2010). Nanoparticles and the immune system. Endocrinology.

[191] Smith D.M., Simon J.K., Baker J.R. (2013). Applications of nanotechnology for immunology. Nat. Rev. Immunol..

[192] Zhao X., Yang K., Zhao R., Ji T., Wang X., Yang X., Zhang Y., Cheng K., Liu S., Hao J. (2016). Inducing enhanced immunogenic cell death with nanocarrier-based drug delivery systems for pancreatic cancer therapy. Biomaterials.

[193] Huang F.-Y., Lei J., Sun Y., Yan F., Chen B., Zhang L., Lu Z., Cao R., Lin Y.-Y., Wang C.C. (2018). Induction of enhanced immunogenic cell death through ultrasound-controlled release of doxorubicin by liposome-microbubble complexes. Oncoimmunology.

[194] Kolar Z., Petrujova J. (1987). Evaluation of the diagnostical significance of lectin histochemistry in breast malignant and benign lesions. Acta Univ. Palacki. Olomuc. Fac. Med..

[195] Lee K., Qian D.Z., Rey S., Wei H., Liu J.O., Semenza G.L. (2009). Anthracycline chemotherapy inhibits hif-1 transcriptional activity and tumor-induced mobilization of circulating angiogenic cells. Proc. Natl. Acad. Sci. USA.

[196] Avramis I.A., Kwock R., Avramis V.I. (2001). Taxotere and vincristine inhibit the secretion of the angiogenesis inducing vascular endothelial growth factor (vegf) by wild-type and drug-resistant human leukemia t-cell lines. Anticancer Res..

[197] Maitani Y., Saito H., Seishi Y., Iwase Y., Yamauchi T., Higashiyama K., Sugino T. (2012). A combination of liposomal sunitinib plus liposomal irinotecan and liposome co-loaded with two drugs enhanced antitumor activity in pc12-bearing mouse. J. Drug Target.

[198] Shi J.F., Sun M.G., Li X.Y., Zhao Y., Ju R.J., Mu L.M., Yan Y., Li X.T., Zeng F., Lu W.L. (2015). A combination of targeted sunitinib liposomes and targeted vinorelbine lposomes for treating invasive breast cancer. J. Biomed. Nanotechnol..

[199] Wang J.L., Xi Y., Liu Y.L., Wang Z.H., Zhang Q. (2013). Combination of targeted pdt and anti-vegf therapy for rat cnv by rgd-modified liposomal photocyanine and sorafenib. Investig. Ophthalmol. Vis. Sci..

[200] Xiao Y., Liu Y., Yang S., Zhang B., Wang T., Jiang D., Zhang J., Yu D., Zhang N. (2016). Sorafenib and gadolinium co-loaded liposomes for drug delivery and mri-guided hcc treatment. Colloids Surf. B Biointerfaces.

[201] Sun W., Wang Y., Cai M., Lin L., Chen X., Cao Z., Zhu K., Shuai X. (2017). Codelivery of sorafenib and gpc3 sirna with pei-modified liposomes for hepatoma therapy. Biomater. Sci..

[202] Abrishami M., Zarei-Ghanavati S., Soroush D., Rouhbakhsh M., Jaafari M.R., Malaekeh-Nikouei B. (2009). Preparation, characterization, and in vivo evaluation of nanoliposomes-encapsulated bevacizumab (avastin) for intravitreal administration. Retina.

[203] Kuesters G.M., Campbell R.B. (2010). Conjugation of bevacizumab to cationic liposomes enhances their tumor-targeting potential. Nanomedicine.

[204] Popov J., Gilabert-Oriol R., Bally M.B. (2017). Unique therapeutic properties and preparation methodology of multivalent rituximab-lipid nanoparticles. Eur. J. Pharm. Biopharm..

[205] Schiffelers R.M., Koning G.A., ten Hagen T.L., Fens M.H., Schraa A.J., Janssen A.P., Kok R.J., Molema G., Storm G. (2003). Anti-tumor efficacy of tumor vasculature-targeted liposomal doxorubicin. J. Control Release.

[206] Pastorino F., Brignole C., Di Paolo D., Nico B., Pezzolo A., Marimpietri D., Pagnan G., Piccardi F., Cilli M., Longhi R. (2006). Targeting liposomal chemotherapy via both tumor cell-specific and tumor vasculature-specific ligands potentiates therapeutic efficacy. Cancer Res..

[207] Gosk S., Moos T., Gottstein C., Bendas G. (2008). Vcam-1 directed immunoliposomes selectively target tumor vasculature in vivo. Biochim. Biophys. Acta.

[208] Volkel T., Holig P., Merdan T., Muller R., Kontermann R.E. (2004). Targeting of immunoliposomes to endothelial cells using a single-chain fv fragment directed against human endoglin (cd105). Biochim. Biophys. Acta.

[209] Kelly C., Jefferies C., Cryan S.A. (2011). Targeted liposomal drug delivery to monocytes and macrophages. J. Drug Deliv..

[210] Kullberg M., Martinson H., Mann K., Anchordoquy T.J. (2015). Complement c3 mediated targeting of liposomes to granulocytic myeloid derived suppressor cells. Nanomedicine.

